# Genomic Rewiring of SOX2 Chromatin Interaction Network during Differentiation of ESCs to Postmitotic Neurons

**DOI:** 10.1016/j.cels.2020.05.003

**Published:** 2020-06-24

**Authors:** Daria Bunina, Nade Abazova, Nichole Diaz, Kyung-Min Noh, Jeroen Krijgsveld, Judith B. Zaugg

**Affiliations:** 1 Structural and Computational Biology Unit, European Molecular Biology Laboratory, EMBL, Meyerhofstrasse 1 Heidelberg 69117, Germany; 2 Genome Biology Unit, European Molecular Biology Laboratory, EMBL, Meyerhofstrasse 1 Heidelberg 69117, Germany; 3 Proteomics of Stem Cells and Cancer, German Cancer Research Center (DKFZ), Heidelberg 69120, Germany; 4 Heidelberg University, Medical Faculty Heidelberg University, Faculty of Biosciences, Heidelberg, Germany; 5 Collaboration for joint PhD degree between the European Molecular Biology Laboratory and Heidelberg University, Faculty of Biosciences, Heidelberg, Germany

**Keywords:** multi-omics, transcription factors, neuronal differentiation, gene regulation, Sox2, Atrx, data integration

## Abstract

Cellular differentiation requires dramatic changes in chromatin organization, transcriptional regulation, and protein production. To understand the regulatory connections between these processes, we generated proteomic, transcriptomic, and chromatin accessibility data during differentiation of mouse embryonic stem cells (ESCs) into postmitotic neurons and found extensive associations between different molecular layers within and across differentiation time points. We observed that SOX2, as a regulator of pluripotency and neuronal genes, redistributes from pluripotency enhancers to neuronal promoters during differentiation, likely driven by changes in its protein interaction network. We identified ATRX as a major SOX2 partner in neurons, whose co-localization correlated with an increase in active enhancer marks and increased expression of nearby genes, which we experimentally confirmed for three loci. Collectively, our data provide key insights into the regulatory transformation of SOX2 during neuronal differentiation, and we highlight the significance of multi-omic approaches in understanding gene regulation in complex systems.

## Highlights

• Complex interplay of RNA, protein, and chromatin during neuronal differentiation
• Multi-omic profiling reveals divergent roles of SOX2 in stem cells and neurons
• SOX2 on-chromatin interaction network changes from pluripotent to neuronal factors
• ATRX interacts with SOX2 in neurons and co-binds highly expressed neuronal genes

## Graphical Abstract

**Figure:**
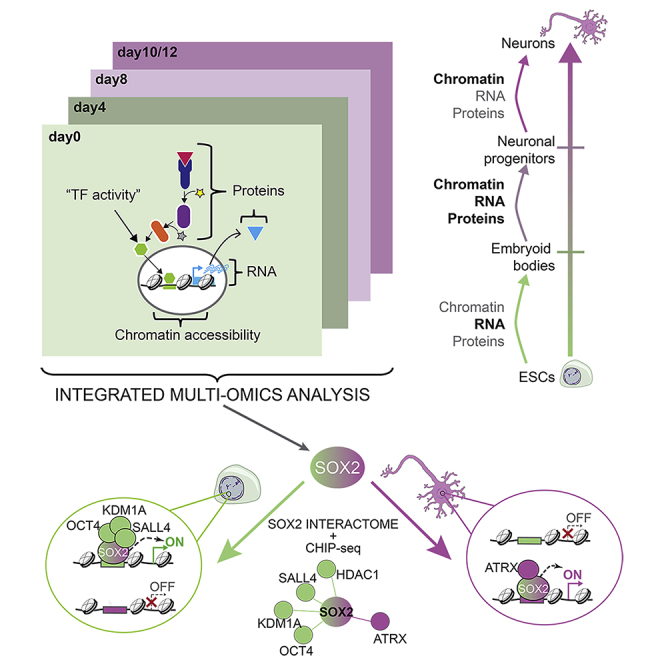

## Teaser

In this work, Bunina et al. profiled transcriptome, proteome, and chromatin accessibility dynamics during differentiation of mouse embryonic stem cells to postmitotic neurons. They revealed extensive associations between molecular layers within and across differentiation time points and uncovered an unexpected interaction of two chromatin-bound proteins, SOX2 and ATRX, in neurons. Their role in regulating neuronal genes was validated by CRISPR. The study highlights the significance of multi-omic approaches followed by specific functional experiments in understanding gene regulation in complex systems.

## Introduction

Cellular plasticity is a fundamental property of cells to dynamically respond to changes in their environment, which is apparent in its most dramatic form during development and differentiation. At the molecular level, differentiation is driven by an intricate process involving multiple regulatory steps to eventually establish a gene and protein expression program that supports the function of the target cell type. Cell-type-specific gene expression is controlled by transcription factors (TFs) through gene regulatory networks (Deplancke, 2009). TFs themselves are typically regulated as downstream effectors of cell signaling pathways, through post-translational modifications, or through induction of their own expression. In addition, their binding to DNA can be regulated by chromatin accessibility (Kaplan et al., 2011, Klemm et al., 2019, Pique-Regi et al., 2011), histone tail modifications (Mikkelsen et al., 2007), and the availability of their interaction partners (Adams and Workman, 1995, Deplancke, 2009), often in a gene or locus-specific manner. These cell-state-specific properties together with TF recognition motifs ultimately determine the TFs target regions and thus their function. Therefore, to understand differentiation at the molecular level, we not only need to understand all levels of TF regulation but also their interactions and mutual interplay.

*In vitro* neuronal differentiation from pluripotent stem cells (Bibel et al., 2007, Germain et al., 2010, Lian et al., 2013) is a paradigm system to study cellular state transitions and their key molecular events and is often used for disease modeling and drug screens (Brennand et al., 2011, Ogawa et al., 2017, Richard and Maragakis, 2015). Different studies have profiled many of the individual molecular layers, such as DNA methylation, histone modifications, chromatin accessibility, gene expression, and proteomics, and have shown that each is essential for understanding the process of differentiation (Frese et al., 2017, Golebiewska et al., 2009, Lomvardas and Maniatis, 2016, Mayran et al., 2019, Tyssowski et al., 2014, Wapinski et al., 2017, Wu et al., 2010, Ziller et al., 2015). However, although these and other studies have provided valuable insights into molecular events during neurogenesis, most of them have focused on one or two regulatory layers. For instance, neural differentiation has been investigated by RNA expression, either alone (Wu et al., 2010) or in combination with chromatin accessibility (Mayran et al., 2019, Zhang et al., 2018), as well as by proteomics (Chaerkady et al., 2009, Song et al., 2019), thereby each providing a partial view of the process and limiting the ability to correlate regulatory principles across multiple levels of regulation. Similarly, an association of proteins with chromatin, as in chromatin immunoprecipitation sequencing (ChIP-seq), is usually determined for a limited number of factors, disregarding potential interaction partners that modulate TF activity. Although various protein interactomes of pluripotency TFs have been characterized (e.g., for OCT4 [van den Berg et al., 2010, Pardo et al., 2010], SOX2 [Lai et al., 2012, Mallanna et al., 2010], and NANOG [Gagliardi et al., 2013] and reviewed in [Huang and Wang, 2014]), they provided no direct evidence that these TFs functionally interact on chromatin.

Here, we applied a hypothesis-free, multi-omic approach to uncover general principles of regulatory rewiring on several molecular levels during differentiation. To this end, we profiled proteome, transcriptome, and chromatin accessibility at 4–6 time points during differentiation of mouse embryonic stem cells (ESCs) to postmitotic glutamatergic neurons. Data integration revealed the pluripotency TF SOX2 as a major regulator of neuronal genes, and we confirmed its abundance in postmitotic neurons. Follow-up ChIP-seq and ChIP-SICAP experiments (selective isolation of chromatin-associated proteins [Rafiee et al., 2016]) revealed extensive genomic redistribution of SOX2 in neurons versus ESCs, which coincided with a drastic change in its chromatin-bound protein interactome. In ESCs, we recover the known pluripotency interactors of SOX2, while in neurons, we identify the chromatin remodeler ATRX as a major SOX2 interactor. ATRX-SOX2 co-localization correlated with an increase in SOX2 binding, enhancer activity, and expression of nearby genes, which we validated at three genomic loci using CRISPR.

## Results

### Multi-omics Factor Analysis (MOFA) Reveals Three Latent Factors Underlying Differentiation Heterogeneity

**Figure 1:**
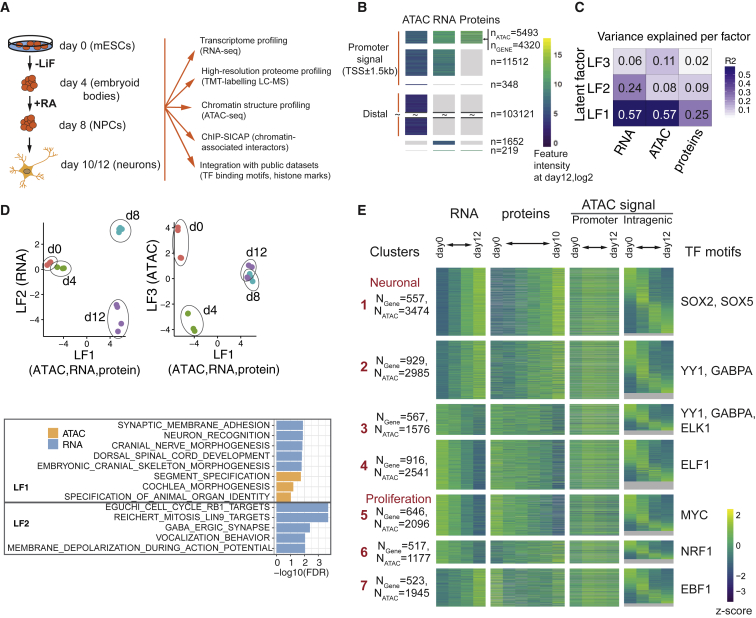
Changes in Proteome, Transcriptome, and Chromatin during Neuronal Differentiation (A) Scheme of neuronal differentiation protocol and experimental set-up (LiF, leukemia inhibitory factor; RA, retinoic acid). (B) Overview of ATAC-seq, RNA-seq, and proteomics data. All except distal ATAC-peaks are aligned by genes. Distal ATAC-peaks are only partially shown due to the high number. n, number of ATAC-seq peaks (or genes if no ATAC-peak is present). (C) Variance explained by latend factors (LFs) identified with MOFA is shown for each dataset. (D) Top: scatterplots of samples projected to LF1 versus LF2 (left), and LF1 versus LF3 (right) are shown. Bottom: a subset of the most enriched GO terms per LF and data type are shown as bar graphs. (E) RNA, protein, and chromatin accessibility data are shown as a heatmap for genes grouped by k-means clustering of log_2_ foldchanges of RNA and protein. Accessibility is shown separately for promoters (1.5 kb from TSS; “Promoter ATAC signal”) and gene body (“Intragenic ATAC signal”). Numbers indicate unique gene IDs (N_Gene_) and intragenic ATAC-seq peaks (N_ATAC_). Top enriched motifs in gene promoters are shown in each cluster (right; full list in Figure S1F). (B and E): genes with multiple promoters and or gene body peaks are shown multiple times. See also Figure S1.

To gain a comprehensive and unbiased overview of the molecular events during neuronal differentiation, we used a differentiation protocol of mouse ESCs to postmitotic neurons (Bibel et al., 2007) that yields about 84%–88% neurons on day 10 (Figure S1A) and performed ATAC-seq, RNA-seq, and MS-based proteomics at several time points (Figure 1A and Table S1). To ensure a pure population of glutamatergic neurons, we verified the absence of expression of marker genes for common contaminant cells in our neuronal cultures (Figures S1A and S1B; STAR Methods). Data were collected for ESCs (day 0), exit from pluripotency after LiF removal (day 2–4), neural progenitors after stimulation with retinoic acid (day 6–8), and mature neurons (day 10–12). Overall, we quantified 117.852 ATAC-seq peaks, 16.940 of which were mapped to a gene promoter (1.5 kb from transcription start site [TSS]), 18.877 gene transcripts, and 4.992 proteins (Figure 1B). Promoter ATAC-seq peaks are more accessible yet less dynamic than distal peaks (Figures S1C and S1G). As expected, the detected proteins represent genes that are relatively highly expressed on RNA level.

We next applied multi-omics factor analysis (MOFA), which infers a low-dimensional representation of multi-omics data in form of latent factors (LFs) (Argelaguet et al., 2018). MOFA identified three LFs that explained a major part of the variance in at least one dataset. LF1 explained the majority of variance in all three layers whereas LF2 and LF3 specifically explain RNA and ATAC-seq data, respectively (Figure 1C). The common factor (LF1) separated early (days 0 and 4) from late (days 8 and 10/12) differentiation, suggesting that drastic changes in cellular processes after neural induction strongly involve all three regulatory layers (Figure 1D). Genes and peaks underlying LF1 were mostly related to general and neuronal morphological transitions (Figure 1D; Table S2). The RNA factor (LF2) captured changes between neuronal progenitors (day 8) and neurons (days 10/12), which were related to neuronal function (synapse and action potential) and to their postmitotic nature (cell cycle). The ATAC factor (LF3) captured changes in early differentiation (days 0 to 4) and was not enriched in any functional terms, likely reflecting the cellular heterogeneity of embryoid bodies. In agreement with this, promoter peaks became generally decompacted at day 4 (Figure S1G).

### RNA and Protein, but Not Chromatin, Show Concerted Changes during Neuronal Differentiation

Next, we investigated the dynamic changes of the individual molecular layers and their relationships. Differential analysis for ATAC-seq and RNA-seq revealed that almost all peaks and genes were differentially accessible and transcribed in at least one comparison (FDR < 5%; 111.200 of 117.852 peaks, and 15.645 of 18.877 genes), thus reflecting the vast epigenetic and transcriptomic changes happening during differentiation. Notably, we only identified 440 differentially expressed proteins (<10% of all detected proteins), 406 of which are also differentially transcribed.

To understand the apparent discrepancy between protein and RNA, we grouped the differentially transcribed genes (that were detected in the proteome) into seven clusters by performing unsupervised clustering (k-means) using log_2_ fold changes of RNA and protein relative to day 0 (= ESCs; n = 4.515; see STAR Methods). Most clusters showed concerted changes between proteins and RNA over time (Figure 1E; clusters 1–5) despite the lack of statistical significance in differential protein expression. The exceptions are genes in clusters 6 and 7, which may be due to post-transcriptional regulation, such as variation in translation rates (Schwanhäusser et al., 2011), or in RNA and protein stability. The latter was corroborated by analyzing protein half-lives from primary mouse neurons (Mathieson et al., 2018), which revealed significantly longer and shorter half-lives of proteins in cluster 6 and 7, respectively (p value = 2.8 × 10^−^³ and 2 × 10^−^⁴, Figures 1E and S1D).

In contrast to the generally concerted behavior of RNA and proteins, promoter accessibility dynamics seems independent of the genes’ expression pattern. This is particularly evident for genes that are upregulated during differentiation and whose promoters are accessible long before they are expressed (clusters 1–2 on Figures 1E and S1G). An exception to this independence is cluster 5, which shows more compaction of promoters at later stages of differentiation in line with decreasing gene expression. Notably, while promoter accessibility was relatively stable, intragenic regulatory elements were highly dynamic during differentiation. Despite the fact that we cannot directly assign functionality to these intragenic accessible regions, these observations suggest that alterations in accessibility of enhancers may play a role in driving gene expression changes during differentiation, while promoter activity may depend on factors beyond accessibility, such as chromatin modifications and TF binding.

Gene ontology (GO) analysis revealed very distinct functions for each cluster, which allowed us to define a “neuronal cluster” (cluster 1) and a “cell proliferation cluster” (cluster 5, Figure S1E). Unexpectedly, one of the most enriched motifs in the neuronal cluster was SOX2, a well-known pluripotency and early neurodevelopment TF (Figure S1F). The proliferation cluster was mainly enriched for motifs of the pluripotency regulator MYC. Notably, even for cluster 6 and cluster 3, which share the downregulation of RNA but show diverging trends on the protein level and differ in their functional enrichment (Figure S1E), we found distinct TF motifs enriched i.e., GABPA/ELK1 for cluster 3 and NRF1 for cluster 6.

In summary, our results show strong correlation between RNA and protein expression dynamics during neuronal differentiation, which allow clustering of genes into functionally distinct groups, whereas promoter accessibility is generally uncorrelated with gene expression.

### Molecular Layers Show Long-Lasting Interactions across Time Points

**Figure 2:**
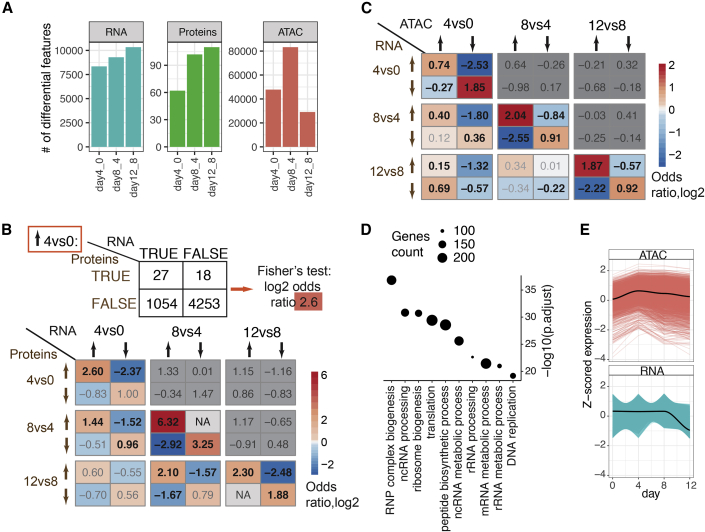
Associations Between Differential Features Across Molecular Layers and Time Points (A) Numbers of differential RNAs, proteins, and ATAC-seq peaks between adjacent time points (day 0 versus 4, 8 versus 4, and 12 versus 8; FDR < 5%.) are shown. (B and C) Associations between differential features across time points are shown as log_2_ ORs according to the schematic in (B) (top). (B): differential RNA and differential proteins, (C): differential promoter ATAC-seq peaks and differential RNA. Adj.p < 0.05 are bold; Fishers’ test; arrows indicate up- or down- regulation at each time point; enrichments contradicting the central dogma of molecular biology (ATAC→RNA, RNA→protein) were not considered (gray boxes). (D) GO terms enriched among genes downregulated at day 12, whose promoters were decompacted already at day 4 (related to the C). Expressed genes with promoter ATAC-seq peak were used as background. (E) Promoter ATAC-seq signal (top) and RNA expression (bottom) dynamics are shown for the genes defined in (D). See also Figure S2.

When assessing each time point individually, we observed an intriguing bias of chromatin changes occurring early in differentiation, whereas transcriptome and proteome changes seemed to increase toward the final differentiation step from neuronal precursors to neurons (day 8 and day 10/12; Figure 2A). Therefore, we next investigated the relationship between regulatory layers across time points. To this end, we tested for pair-wise association of directionalities between RNA and protein and between chromatin and RNA (both up, both down, up-down, and down-up) at the same and subsequent time points using Fisher’s exact test (see schematic in Figure 2B).

Specifically, for RNA and protein, we tested whether genes for which the RNA is upregulated at day X are also upregulated on the protein level at day Y. We then repeated this test for any combination of up and downregulation on RNA and protein level and any day X and Y. As expected, the major associations between RNA and protein expression occur within the same time points (Figure 2B). The only exception to this are transcripts that are downregulated at day 4 with no corresponding downregulation of the proteins, which may suggest that RNA and protein expression during exit of pluripotency is mainly coupled for genes that get induced. Notably, we also observed significant associations between RNA at exit of pluripotency (day 4) and protein at neuronal induction (day 8), both for up- and downregulated genes, and also between RNA at neuronal induction (day 8) and protein in postmitotic neurons (day 10) for upregulated genes. One interpretation of this is that certain RNA patterns established upon the exit from pluripotency and after neuronal induction are coupled to protein expression at the following differentiation state, possibly reflecting a long-lasting effect of RNA expression in neuronal progenitors and postmitotic neurons or a continuous up- or downregulation of the same genes on RNA level. Indeed, we found the same genes being up- or downregulated on RNA level at the subsequent time points (log_2_ odds ratio (OR) between 1.1–2.0, adjusted p value [adj.p] <0.05).

To analyze the associations between chromatin and RNA, we grouped ATAC-seq peaks into promoters (1.5 kb around a gene TSS) and distal elements (all others), and linked them to their closest gene. Similar to the RNA-protein trends, the major associations between chromatin accessibility and RNA occur within the same time points, both for promoters and distal elements, followed by the association between accessibility at day 4 and RNA at day 8 (Figures 2C and S2A). The latter can be interpreted to mean that chromatin changes at the exit of pluripotency are coupled to gene expression at a later stage in neuronal progenitors. For promoters, we found a seemingly contradictory long-term relationship between increase in accessibility at day 4 and decrease in gene expression at day 12 (log_2_OR = 0.69, adj.p < 0.05). These genes were enriched for ribosome biogenesis, translation, and RNA metabolism, all processes that get restructured at exit of pluripotency and again during the transition to the postmitotic state in neurons (Figure 2D). Investigating their RNA expression profiles revealed that they fluctuate across the differentiation, possibly reflecting the metabolic changes at each stage, until they are all downregulated in postmitotic neurons, when also chromatin at their promoter compacts (Figure 2E). Yet, how these molecular events, which are so distant in time, are functionally related, remains open.

### TF Binding Sites Undergo Major Changes in Accessibility during Neuronal Differentiation

**Figure 3:**
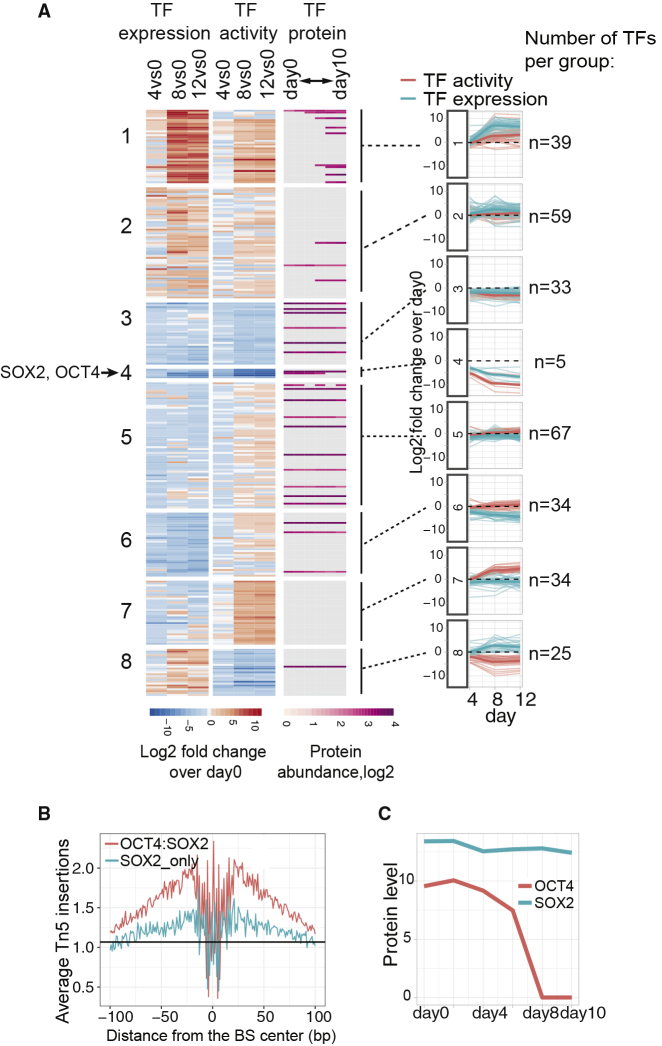
Transcription Factor Binding Sites Undergo Major Changes in Chromatin Accessibility during Differentiation (A) K-means clustering of RNA expression and activity of TFs at days 4, 8, and 12 versus day 0 (log_2_ fold changes) are represented as a heatmap (left) and lineplot (right). Protein abundance (log_2_) is shown for comparison (gray, not detected). Thick lines represent LOESS (local polynomial regression) fit. (B) ATAC signal footprints of the combined OCT4-SOX2 and SOX2-only motif (± 100 bp) at day 0. (C) Protein expression levels (log_2_) of SOX2 and OCT4 during differentiation. See also Figure S3 and Table S3.

To understand the driving forces of the observed molecular patterns, we next investigated the role of TFs in shaping the genome-wide changes in our system. Since not many TFs were quantified using proteomics (43 out 352), we employed our recently developed tool (diffTF) to estimate differential TF activity based on aggregate changes in chromatin accessibility at their binding sites (Berest et al., 2019), see STAR Methods. Overall, we found 296 TFs that significantly changed in activity (Cohens’D > 1) during the differentiation time course (Figure 3A and Table S3).

We clustered the TFs based on their differential activity and change in expression relative to day 0 into 8 major TF groups (Figure 3A; STAR Methods). Among them, we identified a group of “neuronal TFs” (group 1) that strongly increased in activity and expression during differentiation and a group of “pluripotency TFs” that strongly decreased with time (group 4). Notably, for the majority of TFs that were downregulated over time on the RNA level (groups 5–7), we observed a decrease in TF activity. Two TF groups showed strong anticorrelation of TF activity and RNA expression (TF group 7 and 8), which indicates that they act as repressors and compact chromatin when expressed (Berest et al., 2019). Among them are known repressors such as *Zeb1* and *Msx1*.

Among the TFs whose motifs were enriched in the seven gene clusters (Figure 1E), eight were significantly differentially active during neuronal differentiation and appear as transcriptional activators of either developmental genes (in the case of SOX5 and EBF1, groups 1 and 2 in Figure 3A) or pluripotency genes (in the case of MYC and NRF1, group 5 in Figure 3A). A notable exception is SOX2 (group 4), whose motif was enriched in promoters of genes in the neuronal cluster (Figure 1E), yet based on diffTF it showed a decrease in TF activity and RNA expression upon differentiation (Figure 3A, TF group 4). Decreased TF activity and expression of SOX2 is consistent with its role in maintaining pluripotency of the stem cells together with its pluripotency interactors NANOG and OCT4 (group 4 in Figures 3A and S3B). This is further corroborated by the fact that the footprint of SOX2 alone in ESCs shows much weaker signal than the footprint of the OCT4:SOX2 joint motif (Figure 3B). However, while the OCT4 protein disappeared immediately upon exit from pluripotency, SOX2 remained highly expressed at the protein level even in postmitotic neurons (Figure 3C), which we confirmed using immunostaining (Figure S3A).

### SOX2 Relocates from ESC Enhancers to Neuronal Promoters upon Differentiation

**Figure 4:**
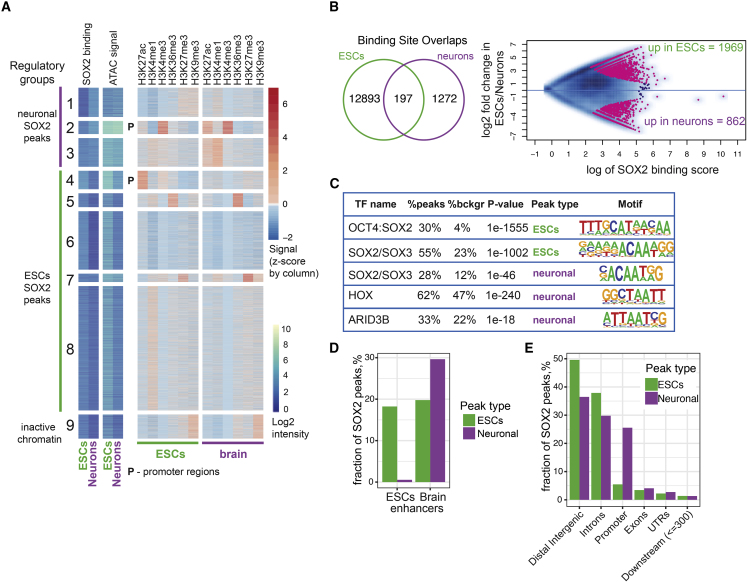
Redistribution of SOX2 Binding Sites during Neuronal Differentiation (A) Regulatory regions occupied by SOX2 (n = 14.362) are clustered into regulatory groups based on k-means clustering of SOX2 binding, ATAC-seq, and ChIP-seq of different histone marks in ESCs and neurons. H3K4me3-marked groups are labeled as promotors (P). (B) Overlap of SOX2 peaks identified in ESCs and neurons displayed as Venn diagram (left). Fold changes of SOX2 binding versus signal intensity are visualized as MA plot. Pink represents differentially bound peaks (FDR < 5%). (C) Top enriched motifs (HOMER tools) are shown for the differential SOX2-peaks in ESCs and neurons. (D) Fractions of SOX2 peaks overlapping known cell-type-specific enhancers are shown (mouse ESCs and E14.5 brain data from Shen et al., 2012). (E) The fractions of ESCs or neuronal SOX2 peaks in different genomic regions are shown. See also Figure S4 and Table S4.

To further understand the cell-state specific role of SOX2, we examined its binding sites in neurons compared to ESCs. To do so, we performed ChIP-seq experiments at days 0 and 10, corresponding to ESCs and postmitotic neurons, respectively, and, to increase power, combined the data with two of the existing datasets in ESCs (Lodato et al., 2013, Whyte et al., 2013) (Figure 4A; Table S4). We identified 14.362 confident SOX2 peaks in the two cell types, most of which overlapped with accessible chromatin regions (11.451 peaks, 81%; see STAR Methods). To characterize the regulatory elements bound by SOX2, we combined our data with publicly available data on active and inactive histone marks (H3K4me1, H3K4me3, H3K27ac, H3K36me3 and H3K27me3, and H3K9me3) from mouse ESCs and forebrain (Shen et al., 2012). Unsupervised clustering of histone marks, ATAC-seq, and SOX2 peaks revealed seven distinct SOX2-bound regulatory groups (Figures 4A and S4A): neuron-specific enhancers (regulatory groups 1 and 3, marked by H3K4me1 and/or H3K27ac), neuron-specific promoters (regulatory group 2, marked by H3K4me3), ESC-specific enhancers (regulatory groups 5, 6, 8), ESC-specific promoters (regulatory group 4), ESC-specific poised regions (regulatory group 7, marked by H3K27me3), and inactive chromatin (regulatory group 9, marked by H3K9me3). Regardless of the regulatory element type, SOX2 binding in neurons always occurred at regions that were already accessible in ESCs (groups 1–3), whereas regions that lost SOX2 binding also lost their accessibility upon differentiation (groups 4–9). We interpret this as evidence for SOX2 not acting as a pioneer factor in neurons, but instead binding to regions that are already accessible through other chromatin binding proteins in ESCs.

Differential binding analysis between ESCs and neurons (Figure 4B) revealed 1.969 and 862 ESCs- and neuron-specific peaks, respectively. In both sets, we found the SOX2/SOX3 motif enriched (Figure 4C); while the ESC-specific peaks were also enriched for the combined OCT4:SOX2 motif, consistent with its role in pluripotency (Aksoy et al., 2013, Merino et al., 2014). Neuronal SOX2 peaks, in contrast, showed an enrichment for the homeodomain HOX motif, found in developmental TFs, and ARID3B motif, an AT-rich interaction domain factor overexpressed in neuroblastomas and interacting with SOX2 in brain tumors (Cox et al., 2013). Genes near neuronal SOX2 peaks were enriched for the GO terms “neuron fate commitment” and “forebrain development” biological processes, whereas genes near ESC-specific SOX2 peaks were enriched for “response to leukemia inhibitory factor” and “histone modification,” consistent with a dual role of SOX2 (Figure S4B).

TFs are often bound to distal regulatory elements acting as tissue-specific enhancers. Using published data of mouse ESCs and brain enhancers (Shen et al., 2012), we observed a higher proportion of ESCs SOX2 peaks overlap ESCs-specific than brain-specific enhancers and vice versa for neuronal SOX2 peaks (Figure 4D). Yet overall, SOX2 peaks in neurons were strongly enriched at gene promoters, whereas in ESCs SOX2 binding was enriched at distal intergenic elements and introns (Figure 4E).

### Chromatin-Associated SOX2 Protein Interaction Network Undergoes Stem Cell- to Neuronal Transition during Differentiation of ESCs

Given the differentiation-induced redistribution of SOX2 from distal, OCT4-co-bound sites to neuronal promoters, we hypothesized that SOX2 may be partnering with a different set of TFs when OCT4 disappears. Therefore, to compare the SOX2 interaction network between ESCs and neurons, we performed ChIP-SICAP (selective isolation of chromatin-associated proteins [Rafiee et al., 2016]) on SOX2, which specifically identifies proteins that interact with DNA-bound SOX2.

**Figure 5:**
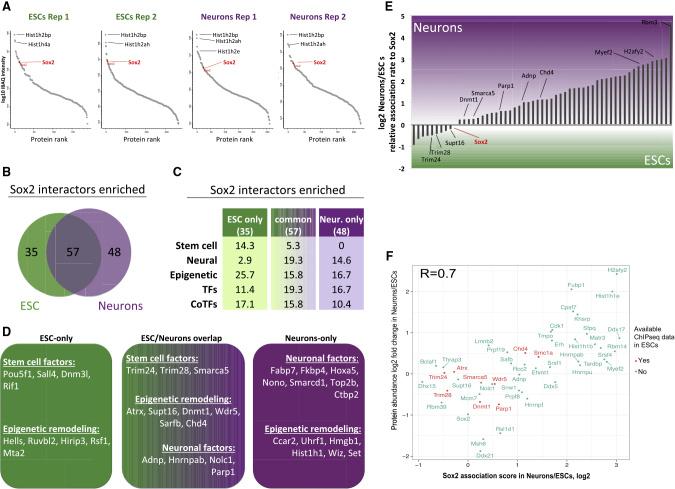
Rearrangement of Chromatin-Bound SOX2 Protein Interaction Network during Neuronal Differentiation (A) Protein enrichment ranking of all SOX2-associated proteins pulled down by ChIP-SICAP. x axis: protein rank, corresponding to the total number of proteins. y axis: iBAQ intensities, log10. (B) Number and overlap between the SOX2-associated proteins in ESCs and neurons. (C) Percentage of different functional groups of SOX2-associated proteins in ESCs and neurons. (D) Selected stem cell, neuronal, and epigenetic factors present in ESCs and neurons alone or in both. (E) Relative SOX2-association rate of overlapping proteins between neurons and ESCs, log2. For details on (C–E) see STAR Methods and Table S5. (F) Scatter plot of the SOX2 association scores from (E) and corresponding protein log_2_ fold changes in neurons versus ESCs. SOX2 interactors with publicly available ChIP-seq data are marked in red.

In all samples and replicates, SOX2 was among the most highly enriched proteins when ranking them based on their protein iBAQ (intensity-based absolute quantification) intensity, indicating a high specificity of the ChIP pull-down (Figure 5A). There were only a few proteins with higher intensity than SOX2, most notably histones, which is expected given their high abundance in chromatin (Figure 5A). To avoid contamination by ubiquitous proteins, only proteins that were either exclusively present in the SOX2 pull downs or displayed at least 4-fold enrichment over the negative IgG control in both biological replicates were included for further analysis.

We identified 92 and 105 proteins that co-localize with SOX2 on DNA in ESC and neurons respectively, of which 57 were found in both (Figure 5B). More than 95% of all identified proteins are nuclear (Table S5), corroborating the high specificity of the method. We found TFs and TF cofactors (CoTFs, as defined in Schmeier et al., 2017) significantly enriched among SOX2 interactors compared to all mouse-protein-coding genes (p = 3.1 × 10⁻⁴, OR = 1.4 for TFs; p = 3.6 × 10⁻^11^, OR = 7.6 for coTFs; Fisher’s exact test), underlining the regulatory essence of the SOX2-centered network (Figure 5C). Another large part of the SOX2 interactors are involved in epigenetic remodeling (e.g., MTA2, HELLS, DNMT1, EHMT1, ATRX, and HMGB; Figure 5D). The high representation of epigenetic factors (20 in ESCs and 19 in neurons) may reflect that the ESC-to-neurons transition is associated with dramatic chromatin and DNA methylation reorganization.

Overall, we observed a marked transition for the pluripotency-related proteins, yet many of the neuronal TFs seem to be already interacting with SOX2 in ESCs (Figure 5C). As expected, OCT4 was among the strongest interactors exclusively interacting with SOX2 in ESCs, along with other stem cell factors SALL4 (known SOX2 interactor) and RIF1 (Figure 5D). The proteins identified as SOX2 interactors in neurons include FABP7, which has been shown to play an essential role for neurogenesis *in vivo* (Watanabe et al., 2007), the topoisomerase TOP2B, which plays a critical role in forebrain development and neuronal migration (Yang et al., 2000), and the CoTF CTBP2, which is a co-activator of retinoic acid signaling (Bajpe et al., 2013) that is essential for neuronal differentiation. The majority of the proteins identified in neurons have not been previously described as SOX2 interactors, possibly owing to the fact that none of the interactome studies focused on differentiated neurons.

Among the SOX2 interactors shared in ESCs and neurons, we determined the preference of the respective interactor in either cell type (Figure 5E; see STAR Methods). We observed a stem cell to neuronal factor transition: the pluripotency-related protein. TRIM28, preferentially bound to SOX2 in ESCs, whereas the neuronal factors ADNP and MYEF2 preferentially bound to SOX2 in neurons. Overall, for most SOX2-interactors, the change in SOX2 association reflects their change in protein expression between neurons and ESCs (Figure 5F; Pearson correlation R = 0.7). There are some exceptions to this (e.g., TRIM24, ADNP, DDX5, or MYEF2), for which the association rate changes much more dramatically than their protein expression level potentially reflecting regulated recruitment to chromatin.

### Distinct Interacting Proteins Co-occupy Genomic Regions with SOX2 in ESCs and Neurons

**Figure 6:**
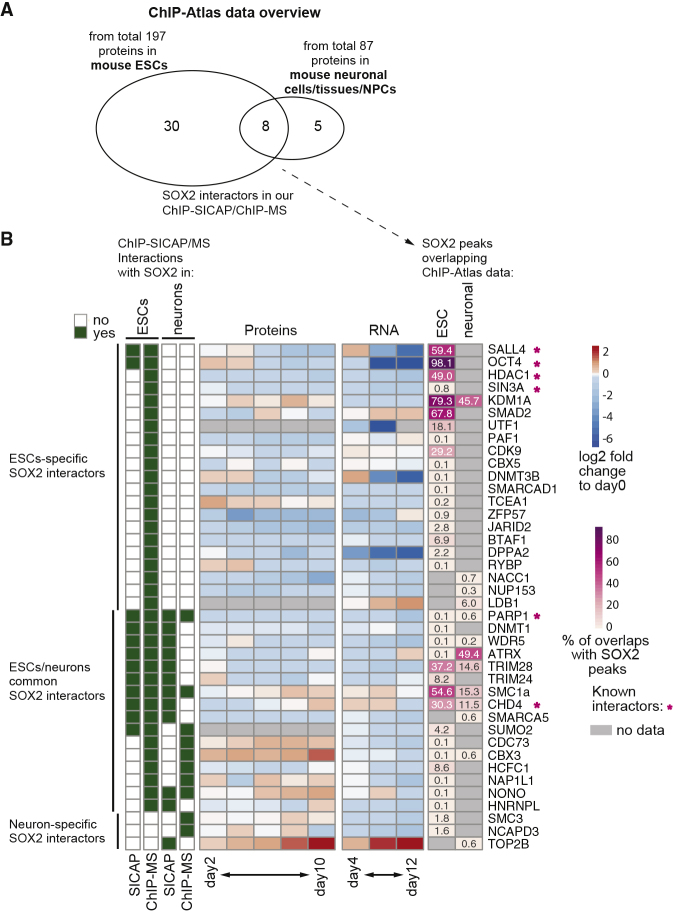
Genomic Co-occupancy of SOX2 and Its Identified Interactors (A) Overlap of SOX2 interactors in ESCs and neurons (based on our SOX2 ChIP-SICAP and ChIP-MS data) with publicly available ChIP-seq data (from ChIP-Atlas) are shown as Venn diagram. (B) SOX2 interactors identified by SICAP or ChIP-MS in ESCs and neurons are shown (left) along with their RNA and protein expression in neural differentiation (middle; log_2_ fold change to day 0) and the fraction of SOX2 peaks that overlap with their binding sites (right). Gray boxes indicate no ChIP-seq data in the corresponding cell type in the ChIP-Atlas database. Stars mark known SOX2 interactors (STRING database (Szklarczyk et al., 2019), experimentally confirmed interactors).

To investigate how the change of its interaction partners may regulate the genomic redistribution of SOX2 throughout differentiation, we next assessed the genomic localization of the SOX2 partners identified above. To do so, we obtained publicly available ChIP-seq profiles (Oki et al., 2018) for mouse ESCs and neurons (or brain). We included both chromatin-associated and nucleoplasmic SOX2 interactors (identified by ChIP-SICAP and ChIP-MS, respectively) in our list to also cover SOX2 complexes that only transiently bind to chromatin. Of the 140 SOX2 interactors, ChIP-seq data were available for 38 and 13 in ESCs and neurons, respectively (Figure 6A).

To assess cell-type-specific co-occupancy, we overlapped the differentially bound genomic locations of SOX2 in ESCs and neurons (1.969 and 862 peaks, respectively), using all available ChIP-seq data in the respective cell type. This revealed that the majority of SOX2 peaks in ESCs were co-occupied with another TF (Figures 6B and S5A; STAR Methods). The major ESC co-localizing factors include not only the known SOX2 partners OCT4 (98%), SALL4 (59%), CHD4 (30%), and HDAC1 (49%) but also KDM1A (79%), SMAD2 (68%), SMC1A (57%), and TRIM28 (37%,). In neurons, we found only ATRX (49%) and KDM1A (46%) substantially overlapping with SOX2 (Figure 6B). Of these, only ATRX interacts with SOX2 on chromatin in neurons based on our ChIP-SICAP data, thus making ATRX the major direct chromatin-associated SOX2-interactor in neurons for which ChIP-seq data were available.

### ATRX Co-occupancy with SOX2 Is Associated with Active Chromatin Marks and High Gene Expression in Neurons

**Figure 7:**
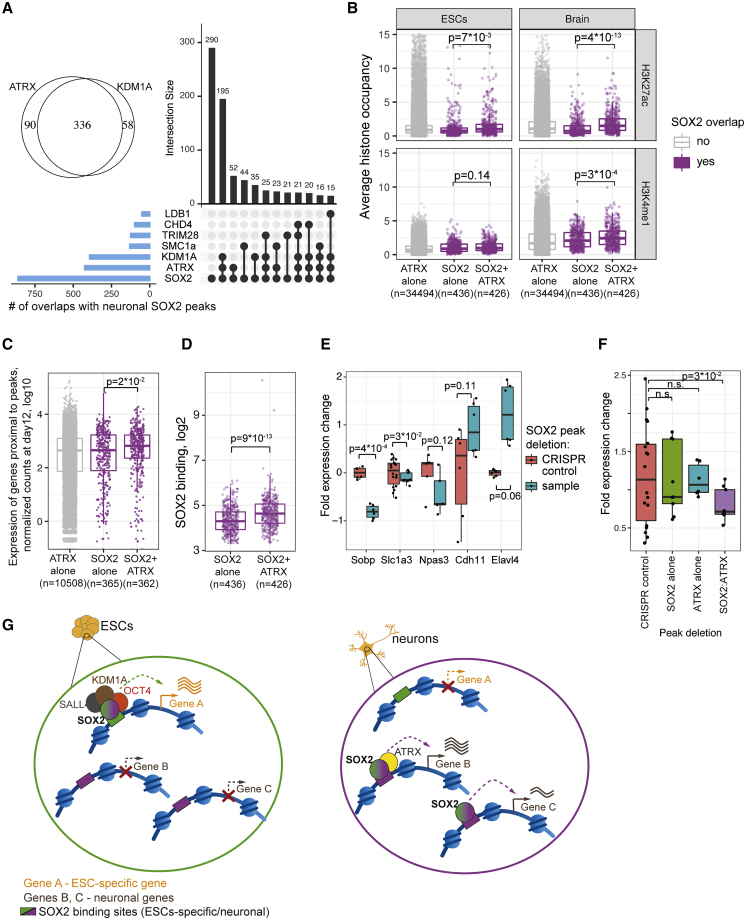
SOX2-ATRX Co-binding Coincides with Increased Enhancer Activity and Is Required for Proper Expression Some Neuronal Genes (A) Intersections of neuronal SOX2 peaks with binding sites of its neuronal interactors are shown as an upset plot. Venn diagram (top left) shows the intersection between the top interactors KDM1A and ATRX binding sites. (B and C) H3K27ac and H3K4me1 occupancy in ESC and brain (B, left and right) and RNA expression in neurons (normalized counts; C) are shown for genomic regions bound by ATRX alone, SOX2 alone, or co-bound by SOX2-ATRX. (D) SOX2 binding strength in neurons for peaks bound by SOX2-only or co-bound by SOX2-ATRX. (E) Expression (qRT-PCR) of 5 neuronal genes on day 12 is shown for 5 lines, in which a SOX2-ATRX co-bound enhancer in the respective genes was removed (blue) normalized to CRISPR control cell lines (red). Each box summarizes at least 2 biological and 3 technical replicates. (F) RNA expression of *Slc1a3* gene (qRT-PCR) is shown for lines in which the indicated regions are removed by CRISPR. (G) Proposed model of SOX2 interaction dynamics during the transition from ESCs to neurons and its possible role in regulating the expression of neuronal genes. All panels: p values are obtained with the Student’s t-test (except for C - Wilcoxon rank-sum test). See also Figure S5 and Table S6.

With clear patterns of a genomic distribution defined, a key question remains to elucidate the function of SOX2 and ATRX interactions in neurons. To address this, we divided the occupied genomic regions into three groups (SOX2-ATRX co-bound, ATRX alone, and SOX2 alone) using the ChIP-seq signals in neurons or brain (see STAR Methods). We compared the levels of histone modifications, marking the activity status of the regulatory regions (enhancer mark H3K4me1 and active mark H3K27ac) available in the ENCODE data from ESCs and mouse frontal cortex. Both H3K27ac and H3K4me1 signals derived from the mouse frontal cortex were significantly increased in the SOX2-ATRX co-bound regions, compared to the SOX2-only regions, while the same groups in ESCs showed only modest (H3K27ac) or non-significant differences (H3K4me1; Figure 7B). This let us hypothesize that SOX2 and ATRX interactions in neurons can function as active enhancers.

To test this, we sought to investigate whether the increased levels of H3K27ac and H3K4me1 would affect the expression of nearby genes. Using ChIA-PET data (Bertolini et al., 2019), we assigned the closest TSS to each SOX2 and ATRX peak and compared the expression levels of the genes proximal to SOX2-ATRX co-bound peaks versus SOX2-only peaks. Indeed, the genes proximal to SOX2-ATRX co-bound peaks showed significantly higher expression compared to SOX2-only peaks (Figure 7C). Furthermore, the genes proximal to SOX2-ATRX peaks were enriched in the neuronal gene cluster from Figure 1E (OR = 2.1, p = 1.6e-3, Fisher’s test), which was not observed for SOX2-only peaks (OR = 1.3, p = 0.26). A possible mechanism for the SOX2-ATRX interaction would be ATRX-mediated stabilization of SOX2 binding. In favor of such a mechanism, we observed higher SOX2 occupancy levels at SOX2-ATRX co-bound regions (Figure 7D).

To validate the function of SOX2-ATRX co-bound sites as neuronal enhancers, we used CRISPR-Cas9 and deleted the regions in the introns of five neuronal genes: *Slc1a3, Sobp, Npas3, Elavl4,* and *Cdh11*, all of which are maximally expressed at day 12 (Figure S5B). Deleting the SOX2-ATRX co-bound region caused a decrease in expression in three out of the five tested genes at day 12 of neuronal differentiation (Figure 7E, p value < 0.05 for two out of three genes, each deletion has two independent cell lines) in comparison with CRISPR control that was treated with CRISPR-Cas9 but did not yield any deletion. Deleting the SOX2 or ATRX alone-bound region in one of the tested genes (*Slc1a3*, see Figure S5C) was unable to change the *Slc1a3* expression (Figure 7F). Notably, deleting a SOX2-ATRX co-bound region in highly expressed genes (*Cdh11* and *Elavl4*, ca. 5-fold higher expression than of *Slc1a3* and *Sobp*, Figure S5B) did not reduce their gene expression (Figure 7E). In accordance with this, reanalysis of the RNA-seq data revealed that for highly expressed genes, ATRX-SOX2 co-bound sites were neither correlated with increased gene expression, nor with H3K27ac level (Figures S5D and S5E), while SOX2 occupancy was still increased when co-bound with ATRX (Figure S5F), which is consistent with a stabilizing role of ATRX on SOX2 binding.

Unlike the effect of removal of the SOX2-ATRX co-bound sites in neurons, removal of the SOX2-OCT4 co-bound regions from the introns of *Slc1a3* and other neuronal or developmental genes, such as *Ncoa1* and *Elavl4*, did not yield any significant changes in their expression in ESCs (Figure S5G). This differs from the conclusion of a previous study, which reported a repressive role of SOX2-OCT4 co-bound regions of developmental genes in ESCs, confirmed in two specific genes (*Meis1* and *Mapk4*; (Cinghu et al., 2017). Thus, it remains an open question, to what extent SOX2-OCT4 co-bound sites have a function in developmental genes.

Based on these data, we propose a model of SOX2 genomic redistribution and interactome rewiring during neuronal differentiation (Figure 7G). In ESCs, SOX2 mainly exists in a complex with OCT4, NANOG, SALL4, and other pluripotency factors and is targeted to regions associated with pluripotency maintenance. In neurons, the SOX2 interactors associated with pluripotency are either not expressed or significantly downregulated, thereby releasing SOX2 from the complexes, enabling it to engage in other interactions, which leads to its recruitment to different genomic regions, such as promoters and enhancers of neuronal genes. SOX2-ATRX co-bound enhancer regions fine-tune the expression of neuronal genes, possibly due to the stabilization of SOX2 binding. This model is supported by our chromatin accessibility data, where neuronal SOX2 binding sites are already accessible in ESCs (Figure 4A, regulatory groups 1, 2, and 3), but not yet occupied with SOX2, which may be explained by SOX2 being trapped in pluripotency protein complexes. The redistribution of SOX2 binding after perturbation of SOX2 interaction with OCT4 and other pluripotency factors (either by introducing an OCT4 mutant incapable of binding to SOX2 or by ectopic expression of another interaction partner BRN2) has been previously reported in reprogramming and differentiation systems (Lodato et al., 2013, Malik et al., 2019).

## Discussion

One of the big challenges in developmental systems biology is to understand how gene regulatory networks switch during the cellular transition from pluripotency to differentiated state, and how this transition is coordinated across molecular levels. In this study, we generated high-resolution transcriptomic, protein expression, chromatin accessibility and chromatin-mediated protein interaction data to create a comprehensive view of the regulatory processes underlying neuronal differentiation of pluripotent stem cells. We note that all our assays were performed on bulk cell populations, and some of our observations might be blurred by averaging across several co-existing cell states, especially in such heterogeneous stages as embryoid bodies on day 4.

Our integrative analyses highlight the multi-level effect of retinoic acid induction (day 8 versus 4) on neuronal differentiation, exerted by driving major changes in RNA and protein expression, as well as chromatin accessibility. This is the only stage of the differentiation process at which we observed major involvement of all three molecular layers. In contrast, the exit from pluripotency (day 4 versus 0) seems driven by chromatin remodeling that is still unspecific to any particular lineage, while the transition between neuronal progenitors and neurons (day 12 versus 8) seems driven by transcription of genes related to neuronal function. The temporal resolution in our dataset enabled us to observe the long-lasting effects of both chromatin on RNA expression and RNA on protein expression. While associations between different molecular levels (e.g., RNA on protein expression, chromatin accessibility on RNA expression) were generally strongest at the same time point, we identified long-lasting effects across and within regulatory layers, which seem to prepare the cell for differentiation processes already at the exit from pluripotency. It further revealed that chromatin accessibility at gene promoters is necessary but not sufficient for productive gene expression, which has been reported in other systems such as response to BMP4 signaling in cancer (Ampuja et al., 2017). This could indicate that developmental gene promoters are already primed by pluripotency factors before developmental TFs bind them.

In addition to previously reported differentiation-driving TFs, such as HOX and LIM homeobox (Lhx) family members (Hobert and Westphal, 2000, Pearson et al., 2005), our data identify SOX2 as a potential regulator in neurons, which was unexpected since SOX2 is primarily known for its role as core pluripotency factor and in neuronal progenitor cells (Favaro et al., 2009, Miyagi et al., 2008). The fact that we did not observe BRN2, a previously reported SOX2 interactor in neuronal progenitors (Lodato et al., 2013), among the SOX2 interactors in neurons, corroborated by the very small (19%) overlap between SOX2 ChIP-seq peaks in neuronal progenitors and postmitotic neurons, provides strong evidence for a distinct role of SOX2 in neurons in addition to the described function of SOX2 in neuronal progenitors (Bergsland et al., 2011, Zhang and Cui, 2014).

In ESCs, SOX2 forms a complex with OCT4 to activate genes involved in pluripotency (Ambrosetti et al., 2000, Boyer et al., 2005, Nishimoto et al., 1999, Yuan et al., 1995), which we recapitulate in our SICAP data. In addition to their involvement in maintaining pluripotency, a previous study has reported a mutually suppressive role of SOX2 and OCT4 in cell-fate choice, through a mechanism that involves differential regulation of SOX2 and OCT4 by external cues (Thomson et al., 2011). Thus, one potential mechanism of how SOX2 identifies its target sites in neurons may involve a change in its interaction partners, which is consistent with our observation that the SOX2 interaction network gets substantially rewired between ESCs and neurons.

We identified ATRX as the most prominent SOX2 interaction partner in neurons. ATRX is a chromatin remodeling factor, which mostly localizes to inactive chromatin regions such as pericentromeric heterochromatin (McDowell et al., 1999) and telomeres (Law et al., 2010). Our observations suggest an additional role for ATRX in neurons where its partnering with SOX2 coincided with increased enhancer activity (H3K7ac and H3K4me1) and expression of nearby genes. While it seems unusual that interaction of SOX2 with the heterochromatin-associated remodeler ATRX led to increased gene activation, there have been several recent reports suggesting a role of ATRX in gene activation (Law et al., 2010, Levy et al., 2008, Levy et al., 2015). A remaining question is what keeps those SOX2-ATRX bound promoters and enhancers of neuronal genes accessible in ESCs since neither SOX2 nor ATRX were bound to them in ESCs.

Taken together, our study highlights the importance of multi-omic approaches for an in-depth understanding of complex biological systems and provides key insights into the regulatory transformations and dynamic interactome in the transition from pluripotent stem cells to neurons, which would not be apparent from looking at any one molecular layer alone.

## STAR★Methods

### Key Resources Table

**Table:** 

REAGENT or RESOURCE	SOURCE	IDENTIFIER
**Antibodies**		

Rabbit polyclonal anti-Sox2 for immunofluorescence	Merck-Millipore	Cat#AB5603; RRID: AB_2286686
Goat polyclonal anti-Sox2 for ChIP-seq/ChIP-SICAP	R&D Systems	Cat#AF2018; RRID: AB_355110
Mouse monoclonal anti-ßTubulin III	Abcam	Cat#AB78078; RRID: AB_2256751
Goat polyclonal anti-mouse Alexa Fluor 594	Thermo Fisher	Cat#A11005; RRID: AB_141372
Goat polyclonal anti-rabbit Alexa Fluor 488	Thermo Fisher	Cat#A11008; RRID: AB_143165

**Critical Commercial Assays**		

Nextera DNA Library Prep Kit	Illumina	Cat# FC-121-1030
NEBNext High-Fidelity 2× PCR Master Mix	New England Biolabs	Cat# M0541
NEBNext Ultra II RNA Library Prep Kit for Illumina	New England Biolabs	Cat#E7770L
NEBNext® Ultra II DNA Library Prep Kit for Illumina	New England Biolabs	Cat#E7645L

**Deposited Data**		

Raw and processed ATAC-seq data	This study	ArrayExpress: E-MTAB-8194
Raw and processed RNA-seq data	This study	ArrayExpress: E-MTAB-8197
Raw proteomics data	This study	Proteomexchange: PXD016080
Raw and processed Sox2 ChIP-seq data	This study	ArrayExpress: E-MTAB-8196
Mouse ESCs, H3K4me1 ChIPseq	ENCODE Project	GSM1000121; https://www.encodeproject.org/experiments/ENCSR000CGN/
Mouse ESCs, H3K9me3 ChIPseq	ENCODE Project	GSM1003751; https://www.encodeproject.org/experiments/ENCSR000ADM/
Mouse ESCs, H3K4me3 ChIPseq	ENCODE Project	GSM1003756; https://www.encodeproject.org/experiments/ENCSR000ADL/
Mouse ESCs, H3K36me3 ChIPseq	ENCODE Project	GSM1000125; https://www.encodeproject.org/experiments/ENCSR000CGR/
Mouse ESCs, H3K27ac ChIPseq	ENCODE Project	GSM1000126; https://www.encodeproject.org/experiments/ENCSR000CGQ/
Mouse ESCs, H3K27me3 ChIPseq	ENCODE Project	GSM1000089; https://www.encodeproject.org/experiments/ENCSR000CFN/
Mouse cortex, H3K27ac ChIPseq	ENCODE Project	GSM1000100; https://www.encodeproject.org/experiments/ENCSR000CDD/
Mouse forebrain E16.5, H3K9me3 ChIPseq	ENCODE Project	GSE82631; https://www.encodeproject.org/experiments/ENCSR352NVU/
Mouse forebrain E16.5, H3K9ac ChIPseq	ENCODE Project	GSE82353; https://www.encodeproject.org/experiments/ENCSR014TEJ/
Mouse forebrain E16.5, H3K27me3 ChIPseq	ENCODE Project	GSE82859; https://www.encodeproject.org/experiments/ENCSR658BBG/
Mouse forebrain E16.5, H3K4me3 ChIPseq	ENCODE Project	GSE82453; https://www.encodeproject.org/experiments/ENCSR129DIK/
Mouse forebrain E16.5, H3K4me1 ChIPseq	ENCODE Project	GSE82464; https://www.encodeproject.org/experiments/ENCSR141ZQF/
Mouse forebrain E16.5, H3K36me3 ChIPseq	ENCODE Project	GSE82630; https://www.encodeproject.org/experiments/ENCSR352AWJ/
Gencode mouse genome annotation version M7	ENCODE	https://www.encodeproject.org/files/gencode.vM7.annotation/
Uniprot database release 2015	Uniprot	https://www.uniprot.org/
ChIP-Atlas database of public ChIP-seq data	ChIP-Atlas	https://chip-atlas.org/
Jaspar CORE release 2018	https://academic.oup.com/nar/article/46/D1/D260/4621338 (Khan et al., 2018)	http://jaspar.genereg.net/api/; RRID:SCR_003030

**Experimental Models: Cell Lines**		

Mouse embryonic stem cells 129XC57BL/6J generated from male 129-B13 agouti mice	Laboratory of Kyung-Min Noh	(Gehre et al., 2020)

**Oligonucleotides**		

See Table S6 for oligos used in CRISPR-Cas9 cell lines generation and for RT-qPCRs quantifications		N/A

**Recombinant DNA**		

N/A	N/A	Addgene plasmid #48138

**Software and Algorithms**		

Proteome Discoverer 1.4	Thermo Fisher	https://www.thermofisher.com/de/de/home/industrial/mass-spectrometry/liquid-chromatography-mass-spectrometry-lc-ms/lc-ms-software/multi-omics-data-analysis/proteome-discoverer-software.html
Mascot	MatrixScience	http://www.matrixscience.com/
TopHat2	https://genomebiology.biomedcentral.com/articles/10.1186/gb-2013-14-4-r36 (Kim et al., 2013)	http://ccb.jhu.edu/software/tophat/index.shtml
Bowtie2 2.3.2	https://www.ncbi.nlm.nih.gov/pmc/articles/PMC3322381/ (Langmead and Salzberg, 2012)	http://bowtie-bio.sourceforge.net/bowtie2/index.shtml
Trimmomatic 0.32	https://academic.oup.com/bioinformatics/article/30/15/2114/2390096 (Bolger et al., 2014)	http://www.usadellab.org/cms/index.php?page=trimmomatic; RRID:SCR_011848
Snakemake 5.0	https://doi.org/10.1093/bioinformatics/bts480 (Köster and Rahmann, 2012)	https://snakemake.readthedocs.io/; RRID:SCR_003475
Deeptools 2.5.0	https://doi.org/10.1093/nar/gku365 (Ramírez et al., 2014)	https://deeptools.readthedocs.io/en/develop/; RRID:SCR_016366
Macs2 2.1.1	(Zhang et al., 2008)	https://github.com/taoliu/MACS; RRID:SCR_013291
FastQC 0.11.5	(Andrews, 2010)	https://github.com/s-andrews/FastQC; RRID:SCR_014583
Picard tools 2.9.0	Broad Institute	https://broadinstitute.github.io/picard/; RRID:SCR_006525
Samtools 1.3.1	https://doi.org/10.1093/bioinformatics/btp352 (Li et al., 2009)	http://www.htslib.org/; RRID:SCR_002105
Homer 4.9.1	(Heinz et al., 2010)	http://homer.ucsd.edu/homer/; RRID:SCR_010881
R 3.5.1	https://cran.r-project.org/doc/FAQ/R-FAQ.html#Citing-R	http://www.r-project.org/ RRID:SCR_001905
Bioconductor	https://www.nature.com/articles/nmeth.3252 (Huber et al., 2015)	https://www.bioconductor.org/; RRID:SCR_006442
MOFA 1.2	(Argelaguet et al., 2018)	https://bioconductor.org/packages/release/bioc/html/MOFA.html
DiffBind Bioconductor package 2.10	(Ross-Innes et al., 2012)	https://bioconductor.org/packages/release/bioc/html/DiffBind.html; RRID:SCR_012918
DESeq2 Bioconductor package 1.20	(Love et al., 2014)	https://bioconductor.org/packages/release/bioc/html/DESeq2.html; RRID:SCR_015687
GenomicAlignments Bioconductor package 1.16	(Lawrence et al., 2013)	https://www.bioconductor.org/packages/release/bioc/html/GenomicAlignments.html
clusterProfiler 3.10	(Yu et al., 2012)	https://bioconductor.org/packages/release/bioc/html/clusterProfiler.html
diffTF	https://www.sciencedirect.com/science/article/pii/S2211124719314391 (Berest et al., 2019)	https://difftf.readthedocs.io/en/latest/

### Resource Availability

#### Lead Contact

Further information and requests for resources and reagents should be directed to and will be fulfilled by the Lead Contact, Judith B. Zaugg (zaugg@embl.de)

#### Materials Availability

Mouse embryonic stem cell lines generated in this study are available from the corresponding author on request.

#### Data and Code Availability

• The accession number for the ATAC-seq data reported in this paper is ArrayExpress:E-MTAB-8194. The accession number for the RNA-seq data reported in this paper is ArrayExpress:E-MTAB-8197. The accession number for the proteomics data reported in this paper is ProteomeXchange:PXD016080. The accession number for the SOX2 ChIP-seq data reported in this paper is ArrayExpress:E-MTAB-8196.
• The code used in this study has not been deposited in a public repository because it was not used to generate new tools or workflows and a combination of already available software was used only for data interpretation and visualization. This custom code is available from the corresponding author on request.

### Experimental Model and Subject Details

#### Cell Lines Used in This Study

All experiments were performed using murine embryonic stem cells (ESCs) (129XC57BL/6J) generated from male 129-B13 agouti mice. The differentiation protocol of mouse ESCs to glutamatergic neurons was performed as described in (Bibel et al., 2007). Briefly, ESCs were cultured on feeder-free gelatin-coated plates for 2 passages in ESC medium containing Knockout-DMEM (Thermo Fisher) with 15% ES Cell Qualified EmbryoMax® FBS (Millipore, ES-009-B) and 20 ng/ml LIF (EMBL protein expression facility, Heidelberg) prior to experiments. Differentiation starts upon transfer of 4e6 cells/10 cm non-adhesive plates (Sigma, P9366 Sigma) and removal of LIF from the medium, leading to the formation of embryoid bodies. On days 4 and 6, retinoic acid (Sigma, R2625) at a final concentration of 5 μM was added to the medium. On day 8, the embryoid bodies were dissociated, brought in single-cell suspension, plated on poly-D-lysine/laminin-coated plates and switched to N2 medium containing DMEM high glucose (Thermo Fisher, 11965-092), 1xN2 supplement (Thermo Fisher, 17502048), 1x B27 supplement (Thermo Fisher, 17504044) and penicillin-streptomycin (1:100, Thermo Fisher, 15140-122). The neurons were maintained till day 10 for proteomics/ChIP-seq/ChIP-SICAP experiments and till day 12 for RNA-seq and ATAC-seq experiments. All cell cultures were maintained at 37^°^ C.

Authentication: genome integrity in mouse ESCs was confirmed by RNA-Sequencing and DNA-Sequencing analysis and comparison to the reference genome (mm10).

The purity of the neurons at day 12 of differentiation was assessed by immunofluorescence using an antibody against neuronal marker ß-tubulin III (Figure S1A). We also detected no expression of the marker genes of the common contaminant cell types (astrocytes, glial cells, and oligodendrocytes) in the RNA-seq data at day 12, and beside the expression of glutamatergic markers (Gria1-4) we detected expression of Sst-positive inhibitory neuronal markers, confirming that our neurons are mostly excitatory with a small population of Sst-positive inhibitory neurons (Figure S1B).

### Method Details

#### Replicates

Proteomics and ChIP-seq experiments were performed in two biological replicates. ATAC-seq experiments were performed in 4 biological replicates obtained from 2 independent rounds of differentiation. RNA-seq experiments were performed in 5 biological replicates obtained from 2 independent rounds of differentiation.

#### Data Exclusion

Generated NGS data was subjected to preliminary quality assessments as recommended by the ENCODE consortium, for example using FastQC v.0.11.5 (Andrews, 2010). No samples were excluded from the analyses and all biological replicates showed high reproducibility by correlation of binned genomic coverage data and/or gene/feature counts and principal component analyses using deeptools version 2.3.3 and Deseq2 version 1.20.0.

#### Randomization

No randomization was required for this cell-culture based in vitro study. All tested cell lines were generated from a common parental mouse embryonic stem cell line.

#### Sample Sizes

Sample sizes of all experiments were chosen in agreement with guidelines for the analysis of next-generation sequencing data and to fulfill the requirements of published bioinformatics tools used in this study (n>=2). The number of analyzed genomic features (n) used to generate plots from the NGS data are depicted in the respective figure, or corresponding figure legend.

#### Proteomic Sample Preparation with SP3

Cells cultured in biological replicates were collected at two-day intervals from day 0 to day 10 and subjected to SP3 for proteome isolation and sample preparation (Hughes et al., 2014). Specifically, cell pellets of 1e6 cells from each condition and replicate were reconstituted in 100 μl of lysis buffer (50mM Ambic, 1% SDS, 1X Protease Inhibitor Cocktail (Roche; 05892791001), 10mM TCEP and 40mM CAA) and sonicated for 12 cycles (30/30 seconds on/off) on a Bioruptor Pico (Diagenode). They were heated up at 95°C for 5 min and cooled down for 5 min at room temperature. A 50/50% mixture of Sera-Mag Speed Beads A and B (Fisher Scientific; CAT No. 24152105050250, CAT No. 44152105050250) was rinsed in water on a magnetic stand 2 times and taken up water, in their original volume. 4μl of the bead mixture was added to the samples and immediately afterwards, 104 μl of acetonitrile (ACN) were added. The samples were left for 10 minutes at room temperature, after which they were placed on a magnetic rack and left for another 2 minutes to allow the magnetic beads to settle. The supernatant was removed, the beads were washed on the rack 2 times with 1 ml 70% ethanol and once with 1 ml ACN. The supernatant was removed, the beads were air-dried for 1 minute and taken up in 20 μl TEAB with pH 8.5. Two μg of the proteolytic enzyme LysC were added and incubated for 16 hours at 37°C.

#### TMT Labeling

Upon protein digestion via SP3, the peptide-containing supernatant was transferred to new tubes. 20 μg (in 1 μl) of TMT reagent was added to each sample and left for 30 min at room temperature. The same amount was added again and left for 30 min at room temperature. 1 μl of quench mix (50mM ammonium bicarbonate and 10mM Lysine) was added to each simple and incubated for 5 minutes. 2 μl of bead mix (see preparation above) was added to each sample and ACN was added up to a final percentage of 95% and incubated for 10 minutes. The beads were put on a magnetic rack for 2 minutes, the supernatant was removed and the beads were washed with 100% ACN. The supernatant was removed and the beads were reconstituted in 4% DMSO in water and placed on magnetic rack. Finally the supernatant was taken in fresh tubes and formic acid was added to a final percentage of 0.1%.

#### Peptide Fractionation

TMT-labeled peptide samples were fractionated with 1,200 Infinity HPLC system (Agilent), using a Gemini C18 column (Phenomenex). A 60 minute gradient was used, which progresses linearly from 0 to 35% ACN in 20 mM ammonium formate, pH10. The flow rate was set at 100μl/minute. Peptide elution was detected via UV detector at 254 nm. 33 fractions were collected and pooled into 11 fractions

(combination strategy: fraction 1, 12 and 23; 2, 13 and 24 etc.).

#### Liquid Chromatography and Mass Spectrometry

Mass spectrometry was performed on an Orbitrap-Fusion Quadrupole-Linear-Ion Trap-Orbitrap hybrid mass spectrometer (Thermo Fisher) coupled to EASY-nLC system (Thermo Fisher). The samples were loaded onto a 100 μm x 2 cm Acclaim Pepmap RSLC trap column (5μm particles, 100Å pores, C18) in 100% solvent A (0.1% formic acid in water, ULCMS Grade, Biosolve) and eluted onto a 75 μm x 50 cm (2μm particles, 100Å pores, C18) Acclaim Pepmap RSLC analytical column by a gradient from 3% solvent B (0.1% formic acid in 80% acetonitrile and 19.9% water, ULCMS Grade, Biosolve) to 50% solvent B in 86 minutes at a flow rate of 300 nl/min. Eluting peptides were analyzed by electrospray using a 10 μm Picotip coated fused silica emitter (New Objective) and a Nanospray-Flex ion source (Thermo) connected to an Orbitrap-Fusion Quadrupole-Linear-Ion Trap-Orbitrap hybrid mass spectrometer (Thermo Fisher). The Orbitrap was operated in positive mode generating profile spectra at a resolution of 60.000 FWHM, AGC target was 1x106, maximum injection time 50 ms. The mass spectrometer was set to data-dependent mode of acquisition (top speed) and the most intense ions (threshold 5x103) were selected for HCD-fragmentation using nitrogen as a collision gas (33% HCD collision energy) by the Quadrupole (1.6 m/z window) and resulting fragments were analyzed by the Linear-Ion-Trap set to rapid scan rate, first mass 120 m/z, an AGC Target of 1x104, a maximum injection time of 50 ms and data type to centroid. Selected ions were excluded for reselection 60 (146 min gradient) seconds with a window of 20 ppm.

#### Proteomics Data Analysis

For the whole proteome analysis, MS spectra were analyzed using Proteome Discoverer 1.4 (Thermo Fisher). Proteins were identified using MASCOT search engine (Matrix Science) and the Uniprot Mus Musculus database (release 2015).

The proteomics data were further filtered (to remove non-unique peptides) and VSN normalized (within-sample data centering and scaling). Replicate dependency was removed using sva R package and missing values were imputed using a “missing at random” method. Differential analysis was performed using the limma R package and the protein log_2_ fold change values relative to day 0 were obtained from the linear fit of the imputed data (related to Figure 1E). Significantly differentially expressed proteins were obtained using limma and DEP R packages, using all pairwise combinations of differentiation time points as contrasts and p-adjusted threshold of < 0.05 (related to Figure S1D; and see Table S1).

Proteome ^∗^.raw data from the ChIP-SICAP and ChIP-MS experiments was searched against Uniprot Mus Musculus (release 2017_08) with MaxQuant 1.5.1.2. The proteomics data was further processed using Perseus software (https://maxquant.net/perseus/). To exclude false-positive interactors of SOX2, the only proteins included in the analysis were either 1) exclusively present in the SOX2 pull downs and not in the IgG pull downs in both biological replicates or 2) displayed at least 4-fold enrichment over the negative IgG control in both biological replicates.

The mass spectrometry proteomics data have been deposited to the ProteomeXchange Consortium via the PRIDE partner repository with the dataset identifier PXD016080.

#### RNA Extraction and Library Preparation

Samples for RNA-seq experiment were collected at days 0, 4, 8, and 12 after LiF withdrawal in 5 biological replicates coming from 2 independent differentiations. RNA was isolated from 800.000-1.000.000 cells using Qiagen RNeasy Mini kit and treated with TURBO DNA-free Kit (Ambion) to remove DNA. RNA quality was verified on an Agilent Bioanalyzer Nano Eukaryote chip. 1ug of total RNA was used for poly-A selection with NEBNext PolyA mRNA magnetic isolation module. Subsequent cDNA synthesis, Illumina Tru-seq adapter ligation, and library preparation were performed with NEBNext Ultra RNA Library Prep Kit for Illumina according to manufacturer's instructions. Library quality and concentration were determined using an Agilent Bioanalyzer DNA1000 chip and a Qubit fluorometer. Libraries were sequenced on Illumina HiSeq2000 in single-end mode.

#### RNA-seq Data Analysis

RNA-seq data were aligned to the mouse genome (mm10) with TopHat2 (Kim et al., 2013) using default parameters. Gene counts were produced using summarizeOverlaps function from the GenomicAlignments R package (Lawrence et al., 2013) and Gencode version M7 annotation of all mouse genes. Pairwise differential expression analysis between all time points was performed using DESeq2 (Love et al., 2014) with p-adjusted value cutoff of 0.05.

#### Gene Ontology (GO) Enrichment Analyses

GO analysis was performed using enrichGO function from the clusterProfiler R package (Yu et al., 2012). Ontology used is “Biological process”, background gene set - all mouse protein-coding genes from the ENSEMBL biomaRt annotation, unless indicated otherwise, p-adjusted cutoff < 0.05.

#### Motif Enrichment Analysis

All motif enrichment analyses were performed with HOMER tools (Heinz et al., 2010). For Figure S1F: motif search was done at +/- 2000bp of the annotated TSS using all differentially expressed RNAs as a background. For Figure 4C: motif search was done at +/- 300bp around the peak summit.

#### ATAC-seq Experiment

ATAC-seq experiment was performed on fresh cells collected at days 0, 4, 8 and 12 after LiF withdrawal according to the protocol (Buenrostro et al., 2015). Briefly, 20.000 cells after harvesting were washed once in cold PBS for 5 min at 500 x g at 4 C. The pellet was gently resuspended in 50ul of cold lysis buffer and spun down immediately at 500 x g for 10 min at 4 C. Transposition reaction mix with the enzyme and transposition buffer from Illumina Nextera DNA Library Preparation Kit was added to the pellet and incubated for 30 min at 37 C. The DNA was purified after transposition using a Qiagen MinElute kit and eluted in 10ul of the provided elution buffer and PCR amplified for a total of 11-13 cycles using barcoded primers from Illumina Nextera XT Index Kit v2. DNA was purified and adapters were removed using Ampure beads (1.4:1.0 beads:sample ratio). The quality and concentration of the eluted libraries were determined using an Agilent Bioanalyzer HS chip and a Qubit fluorometer. Libraries were sequenced on Illumina NextSeq 500 in paired-end mode.

#### ATAC-seq Data Analysis

ATAC-seq data were processed following the steps in (Buenrostro et al., 2013) using the custom Snakemake (Köster and Rahmann, 2012) pipeline. Briefly, reads were trimmed using Trimmomatic (Bolger et al., 2014) and aligned to the mouse genome (mm10) using Bowtie2 with parameters -X 2000 --very-sensitive. The read start sites were adjusted due to the transposon insertion specifics of Tn5. Mitochondrial reads and duplicated reads were removed with Picard tools, peak calling was performed using macs2 with parameters --nolambda --nomodel --slocal 10,000.

To look for differentially accessible regions a consensus peak set using samples at all time points in differentiation was produced using the R package DiffBind (minimum of 2 samples to retain a peak). ATAC-seq signal at the peaks at each time point was calculated using the R package DiffBind (used for the heatmap in Figure 4A).

#### diffTF Analysis

To look for differences in TF activity during differentiation based on ATAC-seq data we used our pipeline called diffTF (Berest et al., 2019) (deposited here https://git.embl.de/grp-zaugg/diffTF). Briefly, this method uses predicted TF binding sites defined by a genome-wide PWM scanner and overlaps these with accessible chromatin in ATAC-seq peaks. As a PWM database source, we used Jaspar CORE 2018 database (Khan et al., 2018), extended TF binding sites by 50 bp in each direction and calculated a fold change in ATAC-seq reads between any two conditions (days 4, 8, 12 vs. day 0), such that the final TF activity value for each TF corresponds to the mean of genome-wide differences in accessibility at the TF’s binding sites between the conditions. Statistical significance was calculated as a Cohen’s distance of the weighted mean differences distribution to the matching distribution calculated for a permuted binding site of the corresponding TF. A list of significantly differentially active TFs with TF activity values, RNA and (if available) protein expression is available as Table S3. Significantly differentially active TFs were split into 8 major groups based on unsupervised k-means clustering of the TF activity and TF expression across differentiation.

#### Immunofluorescence

A modified protocol from Hycult biotech (Version: 04-2010) was used for IF staining. On day 8 after LiF withdrawal embryoid bodies were dissociated and the cells were plated onto poly-D-lysine/laminin coated ibidi 35mm dishes (ibidi, Martinsried, Germany) and kept in N2 media till day 10. On day 10 cells were fixed in 2% paraformaldehyde in PBS for 15 min at RT, washed with PBS, permeabilized with cold methanol for 5 min at -20⁰C and washed again with PBS. The dishes were blocked in 2% BSA in PBS for 30 min and incubated with primary antibodies overnight at 4⁰C (rabbit anti-SOX2 antibody, Merck-Millipore AB5603 and mouse anti-ßTubulin III antibody, Abcam AB78078). Next day the dishes were incubated with secondary antibodies (Alexa488 anti-rabbit and Alexa 594 anti-mouse, both ThermoFisher Scientific) for 30 min at RT, washed and kept in PBS for imaging. Images were acquired with a Nikon Ti-E widefield microscope. For quantification of neuronal ß-tubulin III positive cells nuclei on 2 fields of view with >100 cells each were manually classified into ß-tubulin III positive and negative cells in Fiji.

#### Multi-Omic Factor Analysis (MOFA)

MOFA R package version 1.2.0 was used for the analysis (Argelaguet et al., 2018). ATAC-seq peak counts (4 replicates, 4 time points), RNA gene counts (4 replicates, 4 time points) and protein counts (2 replicates, 4 time points), all variance-normalized, were used as input to the model with default parameters and 3% factor drop threshold. The downstream analysis of the model output was performed with ranked lists of top factor loadings (genes or proteins or ATAC-seq peaks) in each data modality (converted to ensembl gene IDs) as input for gene set enrichment analysis (GSEA (Subramanian et al., 2005)), using mouse gene ontology annotations as a reference list. Each ATAC-seq peak was linked to the nearest gene and these nearest gene lists were used for GSEA.

#### ATAC-seq, RNA-seq and Proteomics Integration

The integration of these three data types was performed based on ENSEMBL gene IDs. Particularly, all transcripts were quantified on gene level, all protein IDs were converted to ENSEMBL gene IDs and all ATAC-seq peaks were associated with the nearest gene (by distance to the annotated TSS) unless stated otherwise. All identified ATAC-seq peaks, gene transcripts and proteins are listed in the Table S1, including ENSEMBL gene identifiers, log_2_ fold changes and significance between each 2 time points tested. The integrated RNA-protein-ATACseq table used for Figure 1E is also included (shows z-scored by row abundances of each data type).

#### Fisher’s Exact Test Associations

Fisher’s exact test for Figures 2B and 2C was performed on combined tables of RNA-proteins (matched by ensembl gene IDs and filtered for genes detected on protein level) or ATAC-RNAs (TSS for Figure 2C: one closest to the TSS ATAC-seq peak was selected, but no further than 1.5kb from TSS; distal ATAC-seq peaks for Figure S2A: each remaining non-TSS peak was assigned to the nearest gene and the table was filtered to only keep peak-gene pairs with both ATAC-seq and RNA signal). Fisher’s exact test was then performed on proportions of RNA-proteins or ATAC-RNA pairs which are differential at the same or subsequent time points; an example of a contingency table is shown in Figure 2B. P-values obtained with all tests within one comparison group (RNA-proteins, ATAC.TSS-RNA, or ATAC.distal-RNA) were corrected for multiple testing using Benjamini-Hochberg approach, resulting in adjusted p-values, and the final results were filtered with adjusted p-value < 0.05 threshold.

#### SOX2 ChIP-seq Experiment

Nuclei of murine ESCs (24 x 10e6 cells/replicate) or differentiated neurons (day 10 after LiF withdrawal, 34 x 10e6 cells/replicate) from 2 biological replicates were extracted and cross-linked with 1.5% formaldehyde for 15 min, lysed and sonicated to solubilize and shear the crosslinked DNA. The resulting nuclear extracts were immuno-precipitated with the SOX2 antibody (R&D Systems, AF2018) overnight at 4°C. Eluted and de-crosslinked DNA was used to prepare sequencing libraries for Illumina using NEBNext Ultra II DNA Library Prep Kit for Illumina. Data were collected using 50 reads single-end mode on HiSeq2000.

#### SOX2 ChIP-seq Data Analysis

Reads were trimmed using Trimmomatic version 0.32 (Bolger et al., 2014), aligned to the mouse genome (mm10) using Bowtie2 version 2.3.2 and duplicated reads were removed with samtools version 1.3.1. Peak calling was performed using macs2 version 2.1.0. Published SOX2 ESCs ChIP-seq data were processed in the same way.

Narrow peaks called by macs2 were extended by 250bp around their middle (to a total width of 500bp) and extended peaks were used as input for all further analyses. DiffBind R package was used to generate a consensus set of peaks in both conditions (only peaks present in at least two samples were kept), for visualization of SOX2 occupancy scores in all peaks for Figure 4A and for differential analysis between the conditions (significantly differential ESC-specific and neuronal peaks with FDR < 0.05, for Figures 4B–4E). The full list of SOX2 ChIP-seq peaks, indicating their differential status, are shown in Table S4.

For the Figure 4A: ENCODE histone modification data (see “Public Data Sets” section) was used to calculate average histone occupancy signal in the SOX2 peaks using the R package GenomicRanges. Our ATAC-seq data from days 0 and 12 was used in a similar way to calculate average chromatin accessibility at the ESC and neuronal SOX2 peaks, respectively.

SOX2 peaks annotation into genomic features (promoters, exons, introns etc.) for Figure 4E was done using ChIPseeker R package (Yu et al., 2015) with 1kb around TSS set for promoter region window.

#### Clustering Analyses

All clustering results shown in this study were obtained by k-means clustering using R base function kmeans with an arbitrary number of clusters in each case, chosen based on visual assessment of the resulting data using a heatmap. In the cases with several visually similar clusters those were merged for further analysis.

In Figure 1E we used a subset of genes, which were differential on the RNA level and for which protein expression was quantified (n = 4515). K-means clustering was done using log_2_ fold changes of RNAs and proteins at days 2/4-10/12 relative to day 0, while the visualization on the figure itself is done using RNA or protein expression values instead (z-scored by row), as well as additional chromatin accessibility signal from the ATAC-seq data. Note that the genes might be shown multiple times if they contain multiple intragenic ATAC-seq peaks (also noted in the figure legend).

#### Public Data Sets

Published SOX2 ChIP-seq raw data from mouse ESCs were downloaded from GEO database under accession numbers GSM1050291 (matching input is GSM1050292) and GSM1082341 (matching input is GSM1082343) and processed as described in the “SOX2 ChIP-seq data analysis” section.

The published ENCODE histone modifications ChIP-seq datasets from mouse ESCs and forebrain that were used in Figures 4A and 7B are listed in the Key Resource Table. We used mm10 assembly ENCODE pipeline processed bigwig files (fold change over control, merged replicates 1 and 2) and calculated average histone occupancy signal in the indicated regions of interest (SOX2 peaks in Figures 4A and 7B, ATRX peaks in Figure 7B) using GenomicRanges R package.

#### ChIP-SICAP

ChIP combined with selective isolation of chromatin-associated proteins (SICAP) was performed as described previously (Rafiee et al., 2016). Briefly, nuclei of 2 biological replicates per condition (ESCs and day 10 neurons) were extracted and lysed from formaldehyde-crosslinked cells (1.5%, 15 min), followed by chromatin shearing and chromatin immuno-precipitation (ChIP) of SOX2 (R&D Systems, AF2018). Next, SOX2-crosslinked DNA was biotinylated using terminal deoxynucleotidyl transferase (TdT, Thermo Fisher) and pulled down using streptavidin-coated beads, thus ensuring the specific isolation of the chromatin-associated SOX2 interactome. As a negative control, the same procedure was performed using an unspecific IgG antibody (Santacruz). Finally, the isolated proteins were subjected to proteolytic digestion and protein identification by LC-MS/MS.

Related to Figure 5C: Percentage of different functional groups of SOX2-associated proteins in ESCs and neurons was manually analysed based on the GO information for each of the proteins in the Uniprot database (UniProt Consortium, 2019) (see full description in Table S5).

Related to Figure 5E: An intensity ratio between each interactor and SOX2 was calculated for both cell types and subsequently the neurons ratio was divided by the ESC ratio, thereby serving as an indicator whether the association of the respective interactor to SOX2 increases or decreases between the two cell types.

#### ChIP-Atlas Data Integration

ChIP-Atlas peak positions data for all available proteins in mouse ESCs, or neurons, or ESC-derived neural cells were downloaded from https://chip-atlas.org/peak_browser. The peak positions were lifted over to the mouse genome version mm10 to match our SOX2 ChIP-seq data using liftOver utility of UCSC Genome Browser. As ChIP-Atlas contains appended data from multiple experiments, the overlapping peaks for each protein were merged into a final non-overlapping set of regions. These regions were overlapped with corresponding SOX2 peaks from our study (ESC ChIP-Atlas data with ESC-specific SOX2 peaks (significant only, 1969 regions), neuronal ChIP-Atlas data with neuronal SOX2 peaks (significant only, 862 regions)) using findOverlaps function from the GenomicRanges R package and a minimum overlap of 1 nt. A list of SOX2 peaks (ESCs-specific or neuronal) indicating overlaps with proteins from ChIP-Atlas is provided in Table S4, including overlaps with neuronal ATRX peaks further used in Figures 7 and S5.

#### CRISPR Experiments

Genomic regions of interest containing SOX2 ChIP-seq peaks (ranging from 340 to 2000 nt) were deleted in mouse ESCs by co-transfecting two Cas9-containing plasmids (pSpCas9-(BB)-GFP, Addgene #48138, and its custom made modification with cloned RFP instead of GFP pSpCas9-(BB)-RFP), each carrying a unique guide RNA (gRNA) to each side of the desired deletion to generate a double-strand break. gRNAs were designed using CRISPR design tool (crispr.mit.edu) to minimize off-target effects, cloned into the above mentioned plasmids and sequenced prior to nucleofection (see the list of gRNAs used in Table S6). 2^∗^10⁶ ESCs were transfected with 2ug each of the GFP- and RFP-containing plasmids using electroporation (Nucleofector kit, Lonza) and plated on MEFs. Cells transfected with both plasmids were selected by single-cell flow cytometry sorting for GFP+/RFP+ cells and sorted into the wells of the 96-well plates containing MEFs. Growing ESCs colonies were screened by qPCR using primers annealing to the regions outside the desired deletion (see the list of primer sequences in Table S6). Putative positive clones were expanded and the deletion was confirmed by PCR and Sanger sequencing. The final positive clones were checked for absence of off-target effects (large duplications, deletions, chromosome arm loss) by RNA-seq as described in (Gehre et al., 2019).

#### Quantitative RT-PCR Experiments

Quantitative RT-PCR analysis was performed in biological quadruplicates. Total RNA was extracted from 800.000-1.000.000 cells with RNeasy Mini kit (Qiagen), cDNA was prepared using High-Capacity cDNA Reverse Transcription Kit (ThermoFisher Scientific, Cat. No. 4368814) according to the manufacturer’s instructions. For each biological replicate qPCR reactions were performed in technical triplicates using Power SYBR Green Master mix (Thermo Fisher Scientific) and StepOne Plus 96-well system according to manufacturer’s instructions. Primer sequences for the tested genes are listed in Table S6. Expression of neuronal genes was normalized to Rpl13 levels and then to the CRISPR control cell lines separately for each differentiation time point. The resulting expression fold changes relative to control cell lines (2e-ddCt values) were used in Figures 7E and 7F.

### Quantification and Statistical Analysis

Statistical analyses were performed using R version 3.5.1. Detailed information regarding statistical tests (Student’s t-test, Wilcoxon rank sum test, Fisher’s exact test) used in this study have been provided in the figure legend or in the respective results or STAR Methods section. Data is presented as datapoints or as boxplots. For all boxplots: the lower and upper hinges correspond to the first and third quartiles, the whiskers extends from the hinge to the largest/smallest value no further than 1.5 ^∗^ IQR from the hinge, line indicates median. Numbers of analyzed genomic regions are depicted inside the figures.

## Supplemental Information

Document S1. Figures S1–S5

Table S1. List of Quantified ATAC-Seq Peaks and Genes on RNA and Protein Level, Indicating Differentially Expressed Genes or Peaks in All Conditions, Related to Figure 1 and STAR Methods

Table S2. Output of the MOFA Analysis, Showing ATAC-Seq Peaks, Genes and Proteins, and Their Weights in Each of the Latent Factors, Related to Figure 2 and STAR Methods

Table S3. A List of Significantly Differentially Active TFs with TF Activity Values, RNA and (if Available) Protein Expression, Related to Figure 3 and STAR Methods

Table S4. SOX2 ChIP-Seq Peaks with Binding Scores and Significance in ESCs or Neurons, Related to Figure 4 and STAR Methods For differential peaks overlaps with co-TFs from ChIP-Atlas are shown, as well as the name of the nearest gene.

Table S5. Manual Annotation of the SOX2 Interactors Identified in the ChIP-SICAP Experiment, Including Their Localization and Functional Classification, Related to Figure 5 and STAR Methods

Table S6. Oligos Used for CRISPR-Cas9 Experiments and qRT-PCR Validations, Related to Figure 7 and STAR Methods

Document S2. Article plus Supplemental Information
