# Supplementary material for: Genomic Rewiring of SOX2 Chromatin Interaction Network during Differentiation of ESCs to Postmitotic Neurons
Source: Cell Syst. 2020 Jun 24;10(6):480–494.e8. doi: 10.1016/j.cels.2020.05.003 (PMC7322528; doi:10.1016/j.cels.2020.05.003)
Supplement: Document S1. Figures S1–S5 [file mmc1.pdf]

Cell Systems, Volume *10*

Supplemental Information

Genomic Rewiring of SOX2 Chromatin Interaction Network during Differentiation of ESCs to Postmitotic Neurons

Daria Bunina, Nade Abazova, Nichole Diaz, Kyung-Min Noh, Jeroen Krijgsveld, and Judith B. Zaugg

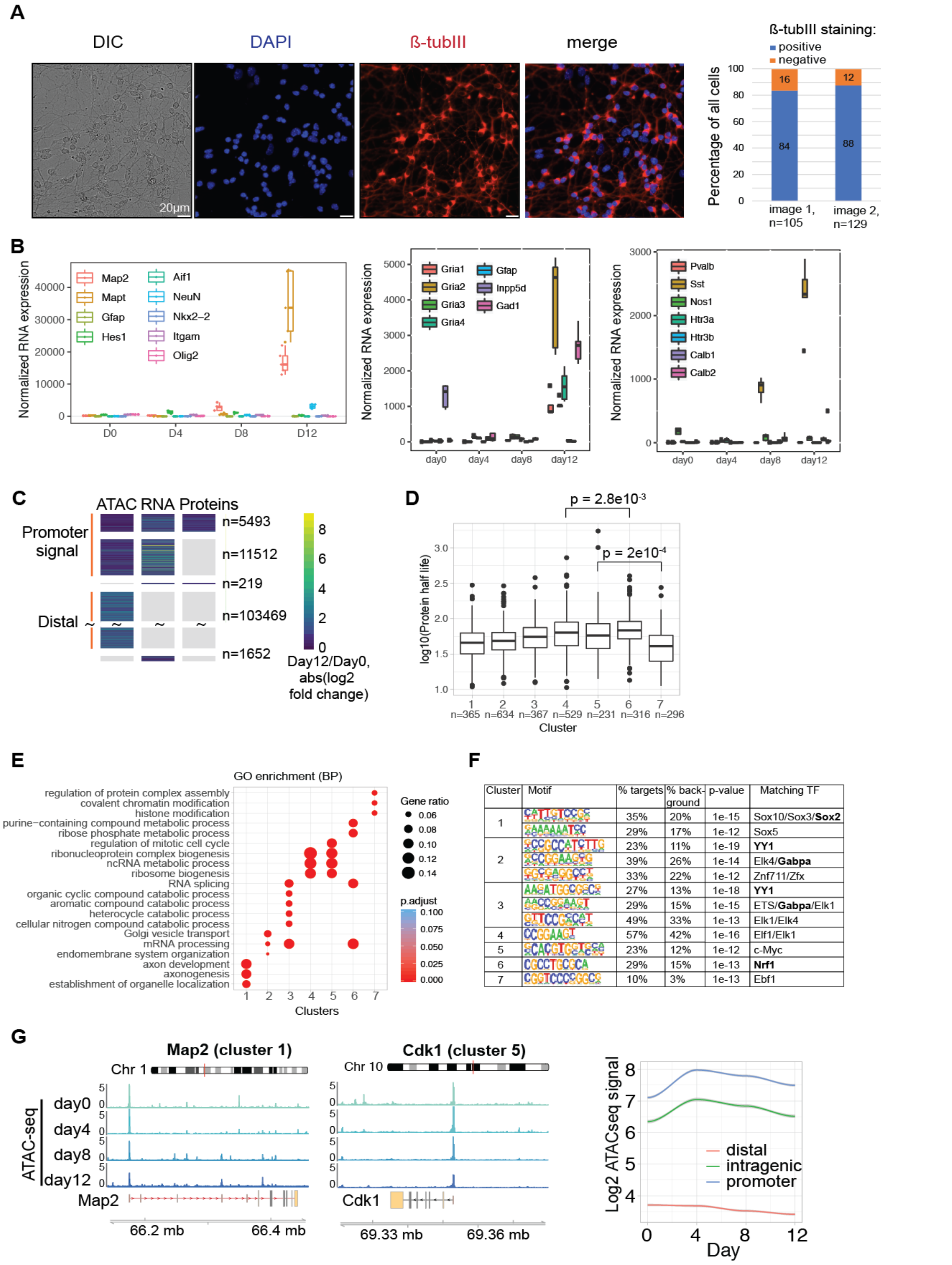

Figure S1, Related to Figure 1. A) Fluorescence microscopy images of day 10 neurons stained with DAPI and neuronal marker ß-tubulin III. Quantification of the percentage of ß-tubulin III positive cells in two images is shown on the right. **B)** Left panel: expression of common neuronal markers (*Map2*, *Mapt*, *NeuN*) and common contaminant cell types: astrocytes (*Hes-1*, *Gfap*), glial cells (*Aif-1*, *Itgam*) and oligodendrocytes (*Nkx2-2, Olig2*). Neither of the

contaminant cell types markers were detected in day 12 neurons. Middle and right panel: Expression of excitatory (middle panel) and inhibitory (right panel) neuronal markers during differentiation. All values are shown as normalized RNA counts per gene. **C)** Same as in Figure 1B but with absolute log2 fold changes between day12 and day 0. **D)** Protein half-lives of the genes in 7 clusters (data from mouse primary neurons (Mathieson et al., 2018); n indicates number of genes). P-values were obtained with a Student's t-test. **E)** GO terms enrichment of biological processes for the clusters shown in Figure 1E. The top significant categories for each cluster are shown. **F)** Motif enrichment analysis in gene promoters (+/- 2kb from transcription start site) by the cluster. Top enriched de novo motifs and matching TFs are shown (>10% of targets with motif, p-value > 1e-12). TFs detected on protein level at any time point are highlighted in bold. **G)** Left: visualization of the example genes from the neuronal cluster (*Map2*) and proliferation cluster (*Cdk1*) showing RNA expression and average ATAC signal in the gene area at each time point. Right: line plots showing ATAC-seq accessibility trends during differentiation at promoter, gene body and distal intergenic regions.

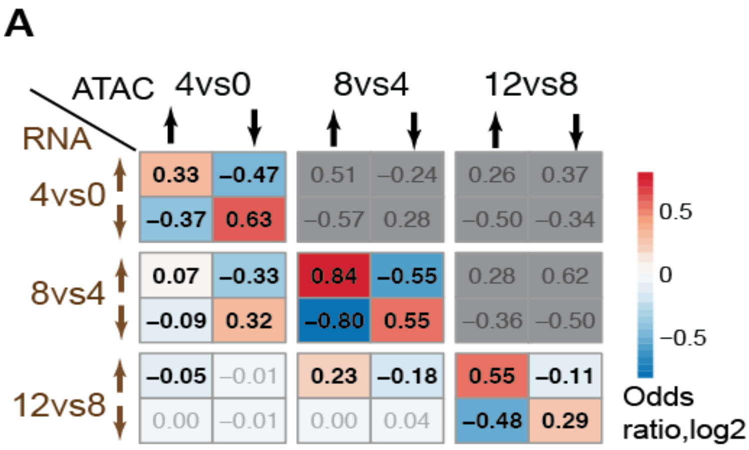

Figure S2, Related to Figure 2. A) Odds ratios (numbers in colored boxes; p-adjusted > 0.05 are greyed out; Fishers' test) of enrichments of differential distal ATAC-seq peaks (>1.5kb from the TSS) across time points (columns) among the differential RNAs across time points (rows). Enrichments contradicting the central dogma of molecular biology were not considered (grey boxes).

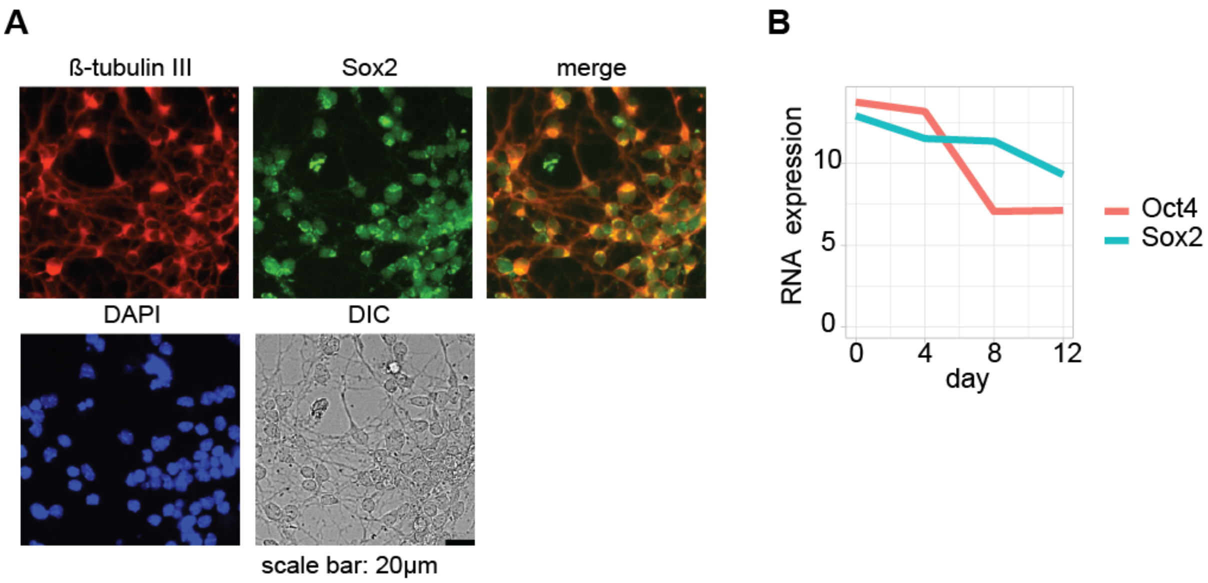

Figure S3, Related to Figure 3. A) Immunostaining of day 10 neurons with antibodies against ß-tubulin III (neuronal marker) and SOX2. **B)** Line plots showing average RNA expression of SOX2 and OCT4 genes during differentiation.

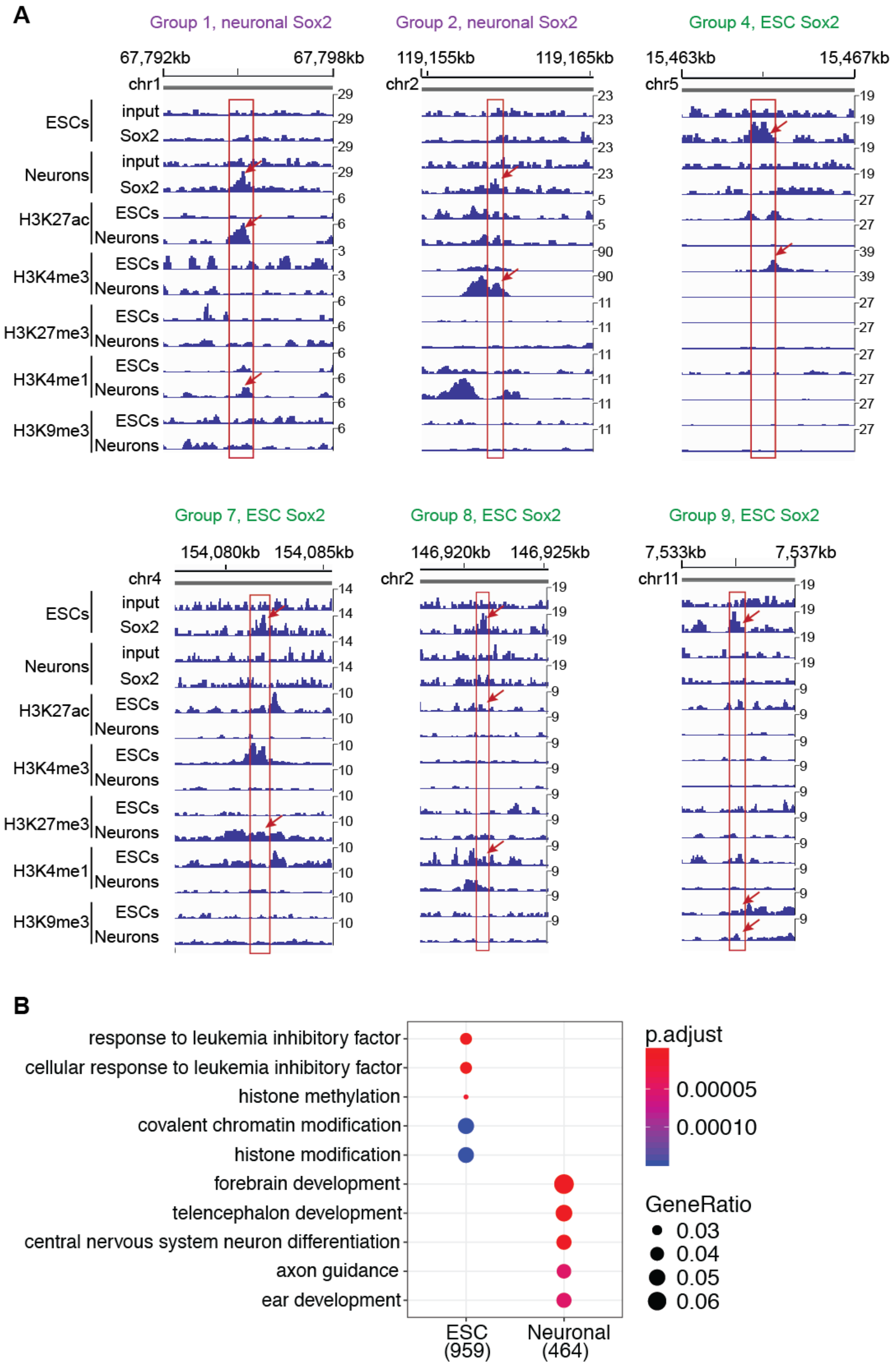

Figure S4, Related to Figure 4. A) Signal tracks of SOX2 peaks picked from regulatory groups displayed in Figure 4A. Arrows indicate relevant SOX2 peak positions (neuronal or ES-specific) and histone marks used in the text to describe the peak regions, e.g. enhancer, promoter or poised. **B)** GO terms enrichment analysis of the genes near significantly differentially bound by SOX2 peaks (neuronal or ESC-specific). Top 5 categories for each group are shown.

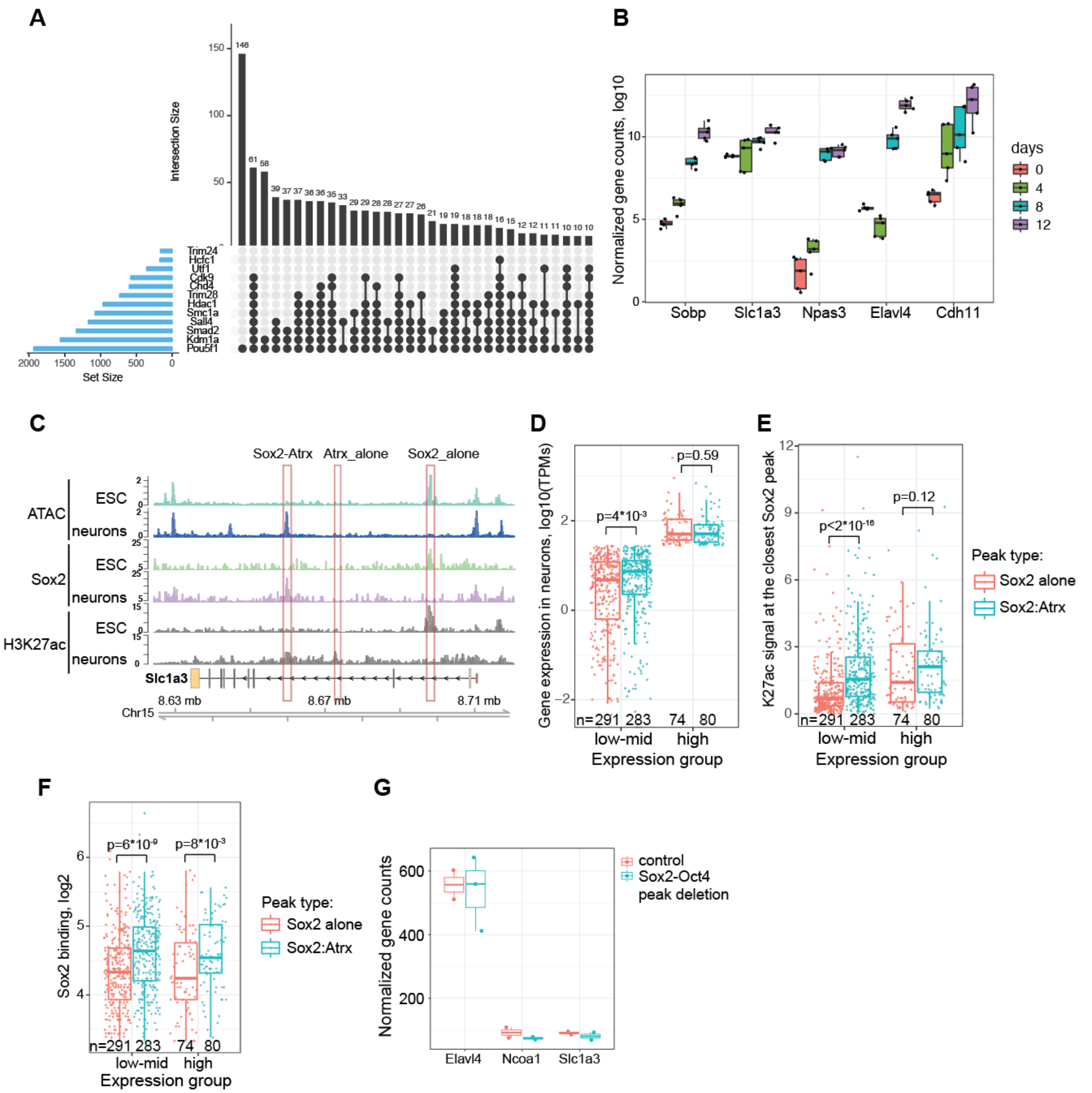

Figure S5, Related to Figure 7. A) Upset Plot showing numbers of SOX2 sites overlapping one or multiple binding sites of SOX2 interactors in ESCs. Horizontal blue bars: total number of SOX2 or SOX2 interactors sites overlapping SOX2 ESCs sites. Vertical black bars: number of SOX2 sites overlapping all SOX2 interactors indicated at the intersection dots. **B)** RNA expression profiles of 5 neuronal genes chosen for validation of the SOX2-ATRX peak function in ESCs. **C)** *Slc1a3* genomic locus, depicting the positions of deleted enhancer regions (co-bound SOX2-ATRX, SOX2-only and ATRX-only bound regions, related to Figure 7F). ATAC-seq, SOX2 and H3K27ac tracks show library-size normalized signal in ESCs and neurons. **D)** RNA expression (TPM counts) of genes proximal to SOX2-only (red) and ATRX-SOX2 co-bound (blue) peaks are shown in neurons at day 12. Genes were split into two groups according to their expression (low-mid: expression < mean TMP; high: expression > meanTPMs) **E-F)** H3K27ac (E) and SOX2 (F) neuronal signal at SOX2-only (red) or SOX2-ATRX co-bound (blue) neuronal peaks. The peaks were split into two groups based on the expression of their proximal genes using the same thresholds as in D). P-values in D), E) and F) were obtained with two-sided Student's t-test. **G)** RNA expression profiles of 3 genes in ESCs upon deletion of SOX2-Oct4 ES-specific peaks (blue boxes), compared the CRISPR control cell lines (red boxes).
